# M7G-Related lncRNAs predict prognosis and regulate the immune microenvironment in lung squamous cell carcinoma

**DOI:** 10.1186/s12885-022-10232-z

**Published:** 2022-11-04

**Authors:** Junfan Pan, Zhidong Huang, Hancui Lin, Wenfang Cheng, Jinhuo Lai, Jiancheng Li

**Affiliations:** 1 Clinical Oncology School of Fujian Medical University, Fujian Cancer Hospital, Fuzhou, 350014 China; 2 The Second Surgical Department of Breast Cancer, Tianjin Medical University Cancer Institute and Hospital, Tianjin, 300060 China; 3 Department of Medical Oncology, Fujian Medical University Union Hospital, Fuzhou, 350001 China

**Keywords:** Lung squamous cell carcinoma, Prognosis, m7G, lncRNA, Tumor immune, Microenvironment

## Abstract

**Background:**

N7-Methylguanosine (m7G) and long non-coding RNAs (lncRNAs) have been widely studied in cancer and have been found to be useful for assessing tumor progression. However, the role of m7G-related lncRNAs in lung squamous cell carcinoma (LUSC) remains unclear. Thus, it is crucial to identify m7G-associated lncRNAs with definitive prognostic value. This study aimed to investigate the prognostic value, correlation with tumor mutation burden, and impact on the tumor immune microenvironment of m7G-related lncRNAs in LUSC.

**Methods:**

LUSC transcriptome data and clinical data were downloaded from The Cancer Genome Atlas, and an m7G-related lncRNA-mRNA co-expression network was constructed using Pearson’s correlation analysis. Cox regression analyses were used to determine a risk model for m7G-associated lncRNAs with prognostic value. The risk signature was verified using the Kaplan–Meier method, receiver operating characteristic curve analysis, and principal component analysis. A nomogram based on risk scores and clinical characteristics was then developed. Gene set enrichment analysis was used for functional annotation to analyze the risk signature. The association among the risk signature, tumor mutational burden, and tumor-infiltrating immune cells was then analyzed. RT-qPCR was used to investigate the expression of 6 m7G-related lncRNAs in LUSC cells. The cytological function of SRP14-AS1 was verified by wound-healing assay and transwell assay.

**Results:**

A total of 293 m7G-related lncRNAs were identifed, 27 candidate m7G-related lncRNAs were signifcantly associated with overall survival (OS). Six of these lncRNAs (CYP4F26P, LINC02178, MIR22HG, SRP14-AS1, TMEM99, PTCSC2) were selected for establishment of the risk model. The OS of patients in the low-risk group was higher than that of patients in the high-risk group (*p* < 0.001). Multivariate cox regression analysis indicated that the model could be an independent prognostic factor for LUSC (HR = 1.859; 95% CI 1.452–2.380, *p* < 0.001). The ROC curve analysis revealed that the AUCs for OS in the 3-, and 5-year were 0.682, 0.657, respectively. GSEA analysis revealed that the risk model was closely related to immune-related pathways. Compared with normal lung epithelial cells, four m7G-related lncRNAs were higher expressed in cancer cells and two were lower expressed, among which knockdown of SRP14-AS1 promoted the proliferation and migration of LUSC cells.

**Conclusion:**

A risk model based on six m7G-related lncRNAs with prognostic value may be a promising prognostic tool in LUSC and guide individualized patient treatment.

**Supplementary Information:**

The online version contains supplementary material available at 10.1186/s12885-022-10232-z.

## Background

Lung cancer is the leading cause of cancer-related death worldwide, with 1.8 million incident cases and 1.6 million related deaths reported annually worldwide [1, 2]. The non-small cell lung cancer (NSCLC) subtype is the predominant type of primary lung cancer, accounting for 85% of all lung cancer cases [3]. NSCLC is divided into adenocarcinoma, squamous cell carcinoma, and large-cell carcinoma. Lung squamous cell carcinoma (LUSC) accounts for approximately 40% of all NSCLC cases. The 5-year survival rate of LUSC patients is closely related to their smoking and economic status [4]. Compared to lung adenocarcinoma (LUAD), LUSC has a poorer clinical prognosis and lacks targeted drugs. Therefore, a more comprehensive understanding of the molecular mechanisms underlying LUSC progression is critical for the development of new therapeutic approaches.

Post-transcriptional modifications play key roles in various physiological and pathological processes [1–3]. To date, more than 170 types of RNA modifications, including N6-methyladenosine (m6A), 5-methylcytosine (m5C), and N7-methylguanosine (m7G) post transcriptional modification have been identified. m7G modification, usually located in the 5 cap and internal positions of eukaryotic messenger RNAs (mRNAs) or within ribosomal RNAs and transfer RNAs (tRNAs) of all species, is one of the most prevalent RNA modifications. Owing to the continuous development of high-throughput sequencing technologies, methods for detecting m7G modifications, including m7G-MeRIP-Seq, m7G-Seq, and m7G-miCLIP-Seq, are constantly being updated. Recent studies have found that m7G methylation also occurs in microRNAs and mRNAs [4, 5] and is critical for maintaining RNA processing metabolism, stabilization, nuclear export, and protein translation [6, 7]. Increasing evidence indicates that abnormal expression of m7G-related genes is closely related to tumorigenesis and progression [8].

In mammals, the most studied m7G regulator is methyltransferase-like 1 (METTL1) [9]. METTL1 normally binds to WD repeat domain 4 (WDR4) and regulates gene modifications [10]. Ying et al. found that METTL1-mediated m7G tRNA modification alters the expression of certain target genes, including EGFR/EFEMP1, and promotes bladder cancer development [11]. The expression levels of METTL1 and WDR4 are elevated in hepatocellular carcinoma (HCC) and are associated with advanced tumor stage and poor patient survival [10]. The expression levels of METTL1 and WDR4 are also significantly higher in lung cancer than in normal lung tissue, and this is closely related to poor prognosis [12]. METTL1 promotes cell proliferation and autophagy through the AKT/mTORC1 signaling axis to promote lung cancer progression [13]. However, some studies have also shown that upregulation of METTL1 impairs the migration ability of A549 cells, thereby inhibiting cell migration [14]. Further in-depth studies are required to reveal the complex functions of METTL1 in LC. Other m7G regulators such as RNMT and RAM are also involved in tumor progression.

Long non-coding RNAs (lncRNAs), which are transcripts over 200 nt in length, are the most important non-coding RNAs [15]. They play key roles in chromatin remodeling, transcription, and post-transcriptional regulation [15]. In addition, the RNA methylation of lncRNAs has been shown to affect cancer progression. In HCC, m5C-modified H19 lncRNA may promote tumorigenesis and development by recruiting G3BP1 oncoproteins [16]. The m6A “eraser” ALKBH5 increases the invasion and metastasis of gastric cancer cells by inhibiting the methylation of lncRNA NEAT1 [17]. Studies of lncRNAs in LUSC have also been widely reported [18, 19]. However, the detailed molecular mechanisms of m7G-related lncRNAs in LUSC treatment and prognosis remain unclear. Many cancer-specific biomarkers have been identified using bioinformatics analysis. However, the association of m7G-related lncRNAs with LUSC prognosis has rarely been reported.

Therefore, this study aimed to investigate the different gene characteristics, prognostic value, correlation with tumor mutation burden and impact on the tumor immune microenvironment of m7G-related lncRNAs in LUSC and provide guidance for the treatment of LUSC. Towards this goal, we screened m7G-related lncRNAs, performed univariate and multivariate Cox regression analyses, identified lncRNAs associated with prognosis, and constructed a prognostic signature to further verify the prognostic value and clinical significance of the model. In addition, the association of the constructed prognostic signature with immune infiltration, somatic mutation, and tumor mutational burden was analyzed.

## Materials and methods

### Data acquisition

The fragments per kilobase of per million (FPKM) of LUSC transcriptome, lncRNA counts data and corresponding clinical data were downloaded from The Cancer Genome Atlas (TCGA) data portal (https://portal.gdc.cancer.gov/). A total of 551 patients, including 502 patients with LUSC and 49 healthy individuals, were evaluated. In total, 403 patients had complete follow-up and clinical data. TMB was defined as the total number of somatic mutations per million bases. The dataset of tumor mutations was also downloaded from TCGA (simple nucleotide variation-masked somatic mutations). The R package “ggpubr” was used to analyze the difference in TMB between the high and low risk groups of the risk model built based on m7G-related lncRNAs. The R package “survminer” was used to analyze the impact of the combined risk model and TMB on overall survival (OS) of patients.

### Screening m7G-related genes and lncRNAs

Forty m7G-related genes were obtained from published articles [6, 20–22] and the gene set enrichment analysis (GSEA) website (http://www.gsea-msigdb.org/gsea/login.jsp) [23], using the keywords “GOMF_M7G_5_PPPN_DIPHOSPHATASE_ACTIVITY,” “GOMF_RNA_7_METHYLGUANOSINE_CAP_BINDING,” and “GOMF_RNA_CAP_BINDING.” lncRNAs were screened from 551 patients with LUSC using Strawberry Perl (version 5.30.0.1). The “limma” package was used to filter m7G-related lncRNAs. The criteria for filtering using Pearson’s correlation analysis were |Pearson R |> 0.4 and *p* < 0.001. Univariate Cox regression analysis was performed to identify prognostic m7G-related lncRNAs using the Kaplan–Meier “survival” package with a cutoff value of *p* < 0.05.

### Constructing the prognostic risk model of m7G-related lncRNAs

TCGA expression and clinical data files were used to investigate the predictive utility of m7G-related lncRNAs in clinical prognosis. Multivariate Cox analysis was used to establish the risk scores, calculated using the following formula:

$$Risk score=coef \left(\mathrm{lncRNAn}\right) \times expr \left(\mathrm{lncRNAn}\right)$$

where coef(lncRNAn) and expr(lncRNAn) represent the survival correlation regression coefficient and the expression value of each m7G-related lncRNA, respectively.

### Evaluation of the risk model of six m7G-related lncRNAs

The patients were divided into the high- and low-risk groups based on the median risk score. Kaplan–meier survival analysis was performed to estimate survival differences between the two groups using the survminer R package. Prognostic analysis using univariate and multivariate Cox regression analyses and the survival R package were used to determine whether clinical characteristics (age, gender, TNM stage) and risk scores could be used as independent prognostic variables. Using rms and survival R packages, age,T stage and risk score were used to create a nomogram for the 1-, 3-, and 5-year OS. Principal component analysis (PCA) was used to perform efficient dimensionality reduction, pattern recognition, and exploratory visualization of total gene expression profile, m7G-related lncRNAs, and risk model lncRNAs expression profiles. The “limma” and “scatterplot3d” packages to perform this process. The receiver operating characteristic (ROC) curve uses the survival R package. Calibration curves use the survival, regplot, and rms R package. Concordance Index curve using survival, rms, pec R package. The ROC curve, calibration curves and C-index curve were used to test the validity of the model.

### Cell culture and quantitative real-time PCR (RT-qPCR)

LUSC cells (H226, SK‐MES‐1) and a normal lung epithelial cell line (BEAS-2B) were purchased from the Procell (Wuhan, China). All cells were cultured in DMEM (meilunbio, Dalian, China) supplemented with 10% fetal bovine serum (Hyclone, Logan, UT, United States). Cells were maintained at 37 °C, 5% CO2.

RNAs were isolated using TRIzol reagent (Invitrogen, USA), Reverse transcription was done using PrimeScriptTM RT reagent Kit (Takara, Japan). SYBR Green PCR Master Mix (Takara) was employed for quantitative PCR on StepOnePlus System (Applied Biosystems).Fold change of gene level was determined by 2^−ΔΔCT^ method, with GAPDH as normalization. The primer sequences involved in this study were shown in Supplementary Table 1. Each PCR reaction was performed in triplicate.

### RNA interference (RNAi) and plasmid transfection

Small interfering RNAs (siRNAs) targeting the SRP14-AS1 sequence were obtained from hippobio (Chaozhou, China) and transfected into SK-MES-1 cells using Lipofectamine 2000 (Invitrogen, CA, USA). The plasmid were transfected into SK-MES-1 cells. The sequences of siRNA were shown in Supplementary Table 2.

### Gene set enrichment analysis and tumor mutation burden

GSEA was used to analyze the biological functions of the two subgroups. Gene sets with a false discovery rate (FDR) of < 0.25 and normalized *p* value < 0.05 were considered significant. GSEA 4.2.1 was used for enrichment analysis in the Kyoto Encyclopedia of Genes and Genomes (KEGG). Tumor mutational burden (TMB) reflects the frequency of gene mutations in tumor tissue. In this study, the “maftools” and “ggpubr” packages in R software were used to visualize mutational data and TMB, respectively, in the risk groups.

### Analysis of immune cell characteristics

The CIBERSORT algorithm was used to investigate the relationship between the risk scores and 22 types of immune cells and their functions. The TCGA tumor immune cell infiltration file was downloaded from TIMER 2.0 and analyzed using the packages E1071, Parallel, PheATMap, CorrPlot, and Vioplot. The TIMER database (https://cistrome.shinyapps.io/timer/) was applied to the analysis of 6 types of immune cells and risk scores, including B cells, CD4^+^ T cells, CD8^+^ T cells, neutrophils, macrophages, and dendritic cells [24]. Using the TIDE (Tumor Immune Dysfunction and Exclusion) (http://tide.dfci.harvard.edu/) algorithm to predict the immune checkpoint reaction inhibitors in scores between high and low risk group.

### Wound-healing assay

Cell viability was measured using a wound-healing assay. Cells in siRNAs group and control group were cultured in 6-well plates with 1%FBS. When the cell density reached 100%, the cell monolayer was scratched with 10ul pipette tip, and then washed three times with PBS. The distance between the two edges of migrating cells was photographed at 0 h and 24 h using a microscope. All experiments were repeated at least thrice.

### Transwell assay

For the migration assay, approximately 1.5 × 10^4^ cells were placed in 200ul serum-free medium in the upper chamber of the tranwell system. For the invasion assays, the upper chamber was covered with matrigel and placed in a 37℃ incubator for 4 h to allow matrigel to solidify. Approximately 1.5 × 10^4^ cells were placed in 200ul serum-free medium in the upper chamber of the tranwell system. 600 mL RPMI 1640 medium containing 10% FBS was placed in the lower chamber as a chemoattractant. After 24 h of incubation, cells in the upper chamber were removed, and the lower chamber was fixed with formaldehyde and stained with crystal violet. The number of cells was counted using ImageJ software. All experiments were repeated at least thrice.

### Statistical analysis

Pearson correlation analysis was used to investigate the correlation between m7G-related genes and m7G-related lncRNAs. Cytoscape was used to visualize m7G genes and m7G-related lncRNAs. Ggpubr R package was used to analyze the correlation between the expression of six m7G-related lncRNAs and clinicopathological factors. Wilcoxon rank sum test was used to compare the expression levels of lncRNAs between unpaired samples, and wilcoxon signed rank test was used to compare the expression levels of lncRNAs between paired samples.Univariate Cox regression analysis was used to calculate hazard ratios (HR). Multivariate Cox regression analysis was used to determine independent prognostic factors for the risk score. ROC curves were generated to evaluate the specificity and sensitivity of the prognostic model. Strawberry Perl was used to synthesize data matrices. All statistical analyses were performed using the R software (version 4.2.1). The threshold of significance was set at *p* < 0.05.

## Results

### Screening m7G-related lncRNAs with prognostic value

**Fig. 1:**
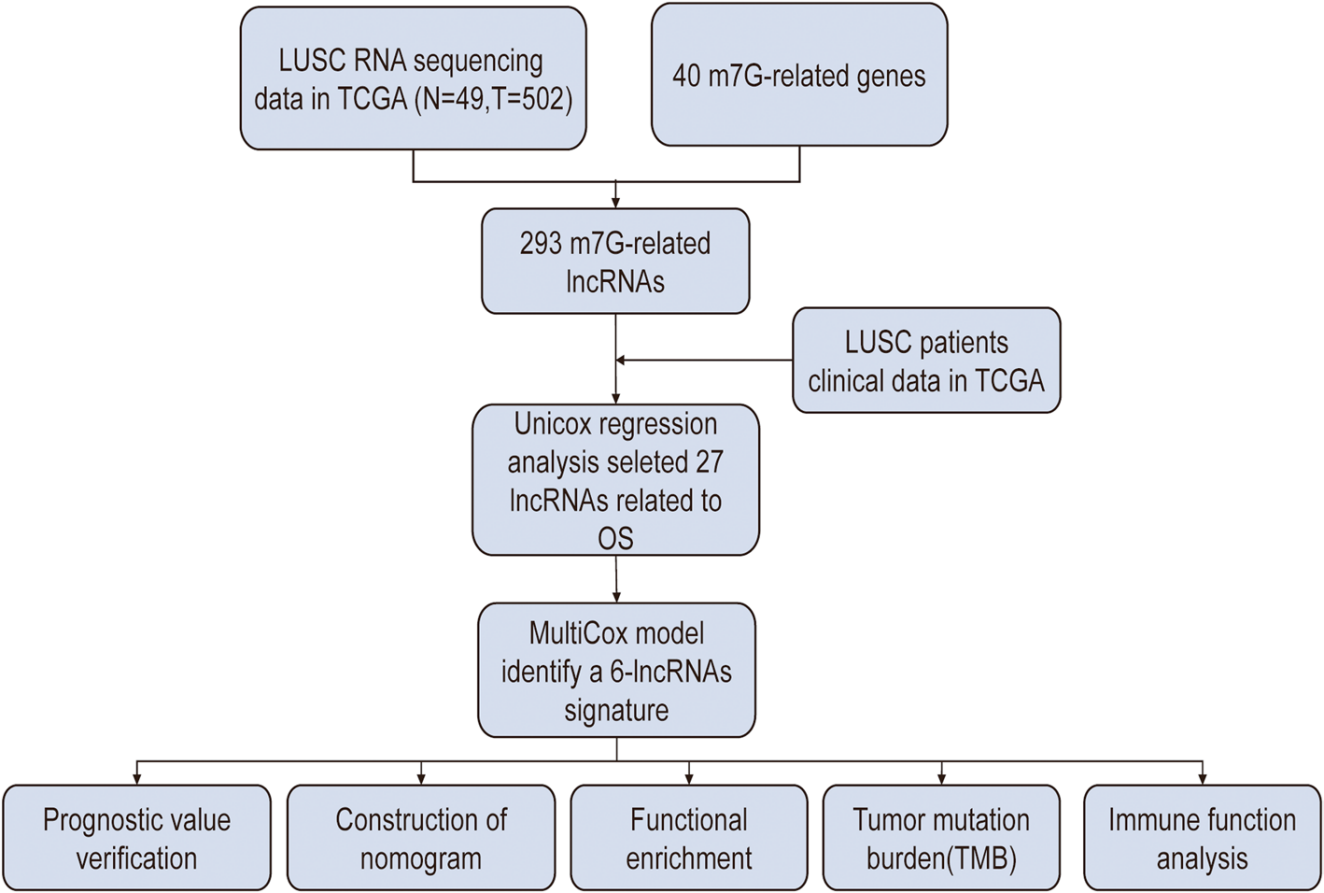
Study workflow. First, m7G-related lncRNAs in LUSC are screened using the TCGA database, and the screened lncRNAs are analyzed using Cox regression to develop a prognostic model of m7G-related lncRNAs. Second, the reliability of the model is verified using various methods. Based on this model, Gene set enrichment analysis (GSEA), tumor mutational burden (TMB) analysis, and immune correlation analysis were performed to determine the potential function of prognostic signatures

**Fig. 2:**
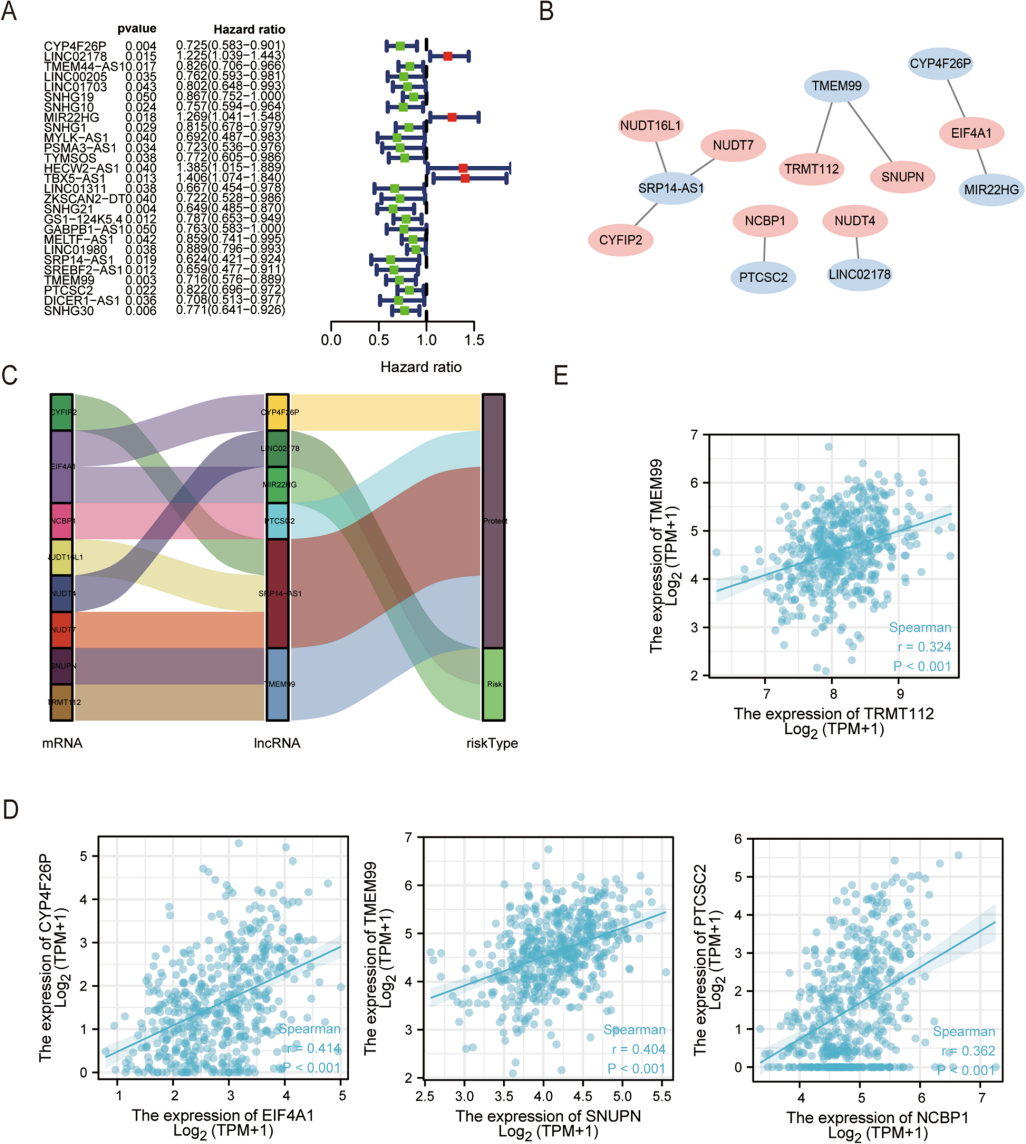
Identification of m7G-related lncRNAs with significant prognostic value in LUSC. **a** In total, 27 lncRNAs associated with prognosis are screened using univariate Cox regression analysis (*p* < 0.05). **b**, **c** Correlation network of prognostic m7G-related lncRNAs and their associated mRNAs. **d**, **e** Co-expression intensity of prognostic m7G-related lncRNAs and their associated mRNAs

The workflow of prediction model analysis was shown in Fig. 1. In this study, we used data from 551 patients with GC from the Cancer Genome Atlas (TCGA) cohort (T = 502, *N* = 49). In total, 293 m7G-related lncRNAs combined with LUSC survival data were identified from TCGA. The correlations between these lncRNAs and m7G methylated genes are summarized in Supplementary Table 3. Subsequently, univariate Cox regression analysis was performed to determine m7G-related lncRNAs with important prognostic values. Among the 27 m7G-related lncRNAs screened (*p* < 0.05) (Fig. 2a), 23 and 4 m7G-related lncRNAs were proven to be high- (HR > 1) and low-risk (HR < 1) factors, respectively.

Multivariate Cox regression analysis was used to screen six lncRNAs associated with prognosis, and the respective coefficients of these lncRNAs were calculated (Table 1).

**Table 1:** The six m7G-related prognostic lncRNAs

ID	Coef	HR	HR.95L	HR.95H	*p* Value
CYP4F26P	0.291726709	0.746972649	0.60359396	0.924409744	0.007301158
LINC02178	0.208888617	1.232307733	1.026935409	1.47875157	0.024722689
MIR22HG	0.176669348	1.193236482	0.9711053	1.466178076	0.092765893
SRP14-AS1	0.453016364	0.635707731	0.429342656	0.941262913	0.023681998
TMEM99	0.224038278	0.799284539	0.635426839	1.005396271	0.055621829
PTCSC2	0.155877204	0.855664256	0.720764796	1.015811709	0.074954294

A co-expression network of m7G-related lncRNA-mRNAs was then constructed, and we detected the highest number of lncRNAs co-expressed with mRNA EIF4A1 (*n* = 2), and the highest number of mRNAs co-expressed with lncRNA SRP14-AS1 (*n* = 3), followed by lncRNA TMEM99 (*n* = 2) (Fig. 2b). We also established a Sankey diagram that showed the relationship among m7G mRNA, lncRNAs and their roles in LUSC (Fig. 2c). Pearson correlation analysis was then used to analyze the expression intensity of related genes in the co-expression network (Fig. 2d, e and Supplementary Fig. 1). m7G-related mRNAs and lncRNAs showed moderate and weak positive correlations. Among them, EIF4A1 and CYP4F26P showed the strongest correlation (*r* = 0.414), followed by SNUPN and TMEM99 (*r* = 0.404), NCBP1 and PTCSC2 (*r* = 0.362), and TRMT112 and TMEM99 (*r* = 0.324).

### Construction of m7G-related lncRNA signature for LUSC

**Fig. 3:**
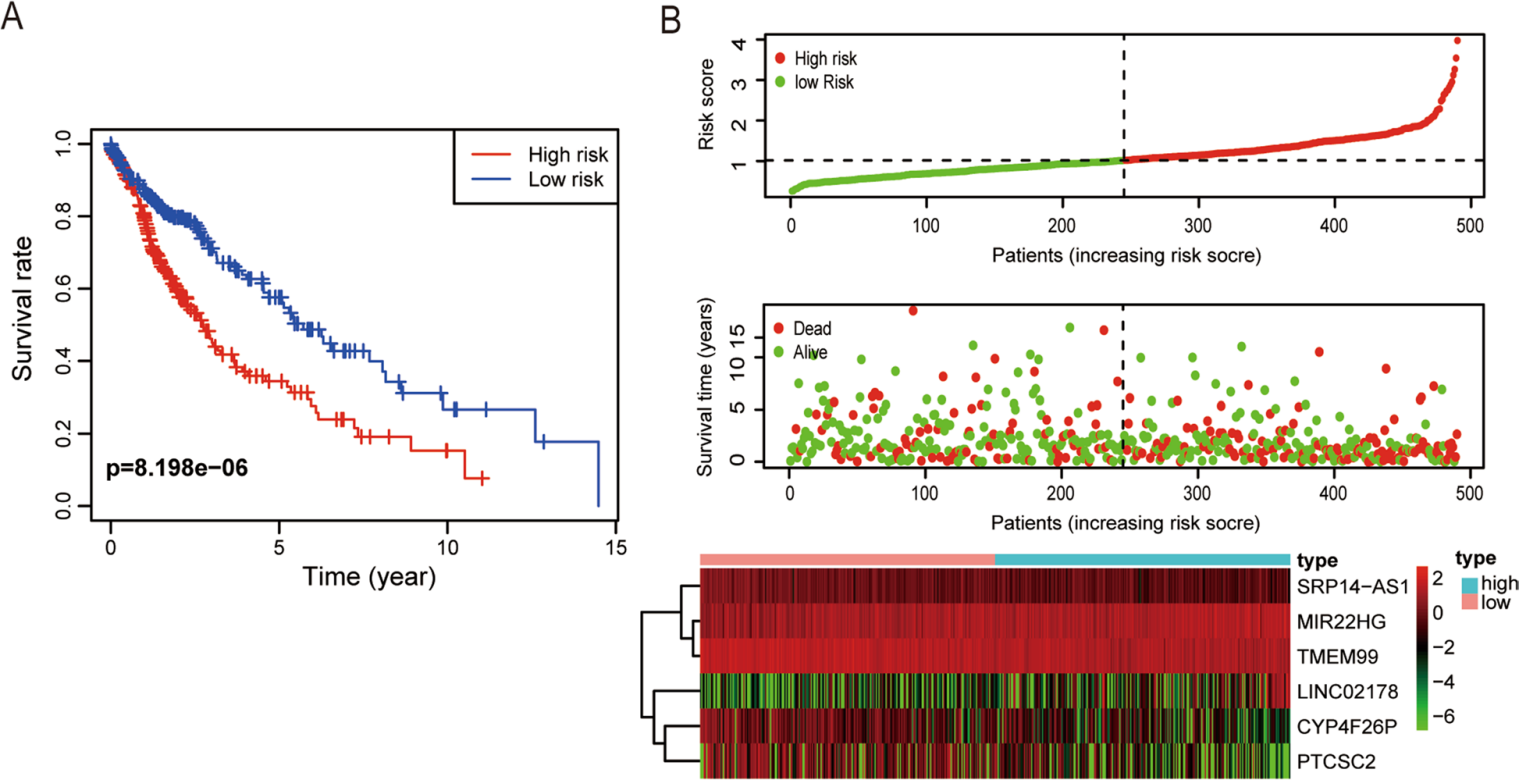
Prognostic value of risk models based on m7G-related lncRNAs. **a** Kaplan–Meier OS curves of the high- and low-risk LUSC patients. **b** Scatter plot of survival status and risk score (X-axis shows the risk score of the LUSC patients; Y-axis indicates the survival time for each patient)

The TCGA-LUSC cohort was divided into the high- and low-risk groups based on the median risk score. Kaplan–Meier survival analysis showed that the OS was lower in the high-risk group than in the low-risk group (*p* < 0.001) (Fig. 3a). Visualization of the distribution of the risk score and survival status showed that the higher the risk score value, the higher was the mortality rate (Fig. 3b). Hence, m7G-related lncRNAs with important prognostic value were identified, and the prognostic value of the signature based on the six m7G-related lncRNAs was determined.

### Correlation between differential expression of m7G-related lncRNAs and clinicopathological variables

**Fig. 4:**
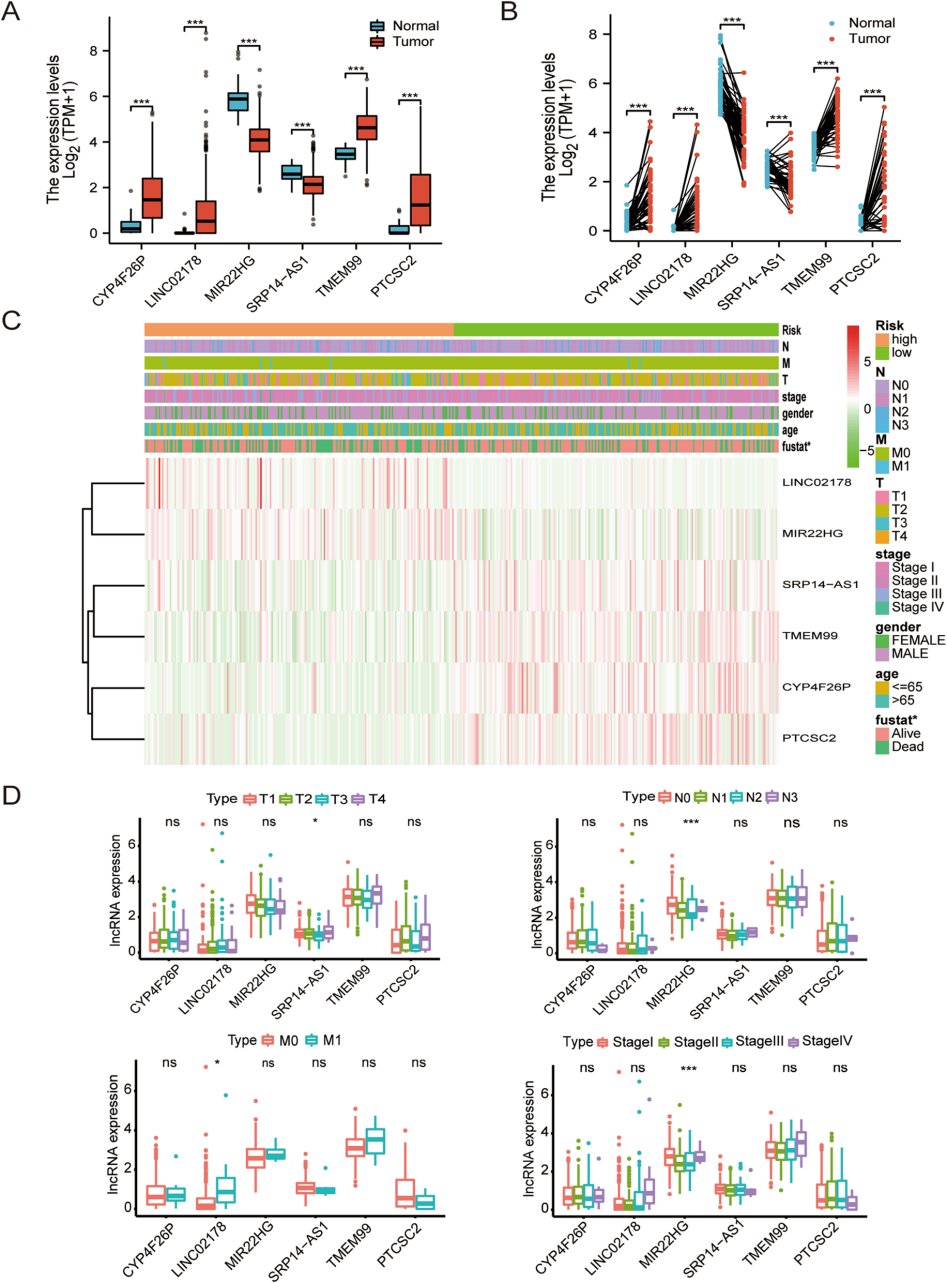
Correlation between the expression of the 6 m7G-related lncRNAs and clinicopathological factors. **a**, **b** Differences in the expression of the 6 m7G-related lncRNAs between LUSC cancer tissues and adjacent normal tissues. **c** Heatmap showing the clinicopathological characteristics of the high- and low-risk groups. **d** Differences in the expression of the 6 m7G-related lncRNAs according to T, N, M, and S stage groups. *p < 0.5, **p < 0.01, and ***p < 0.001. ns, no sense

The expression levels of six m7G-lncRNAs in the tumor and normal samples are shown in Fig. 4a. In unpaired samples, the expression levels of CYP4F26P, LINC02178, TMEM99, and PTCSC2 were higher in cancer tissues than those in adjacent normal tissues (*p* < 0.001), and the expression levels of MIR22HG and SRP14-AS1 were higher in adjacent normal tissues than those in cancer tissues (all *p* < 0.001). Similar results were obtained in the paired samples (Fig. 4b). Using RT-qPCR to further verify at the cytological level, it can be seen that the expression level of CYP4F26P, LINC02178, TMEM99, and PTCSC2 in LUSC cells was higher than that of Beas-2b, and the expression level of SK-MES-1 was higher than that of H226. The expression of MIR22HG and SRP14-AS1 in LUSC cells was lower than that of Beas-2b (Supplementary Fig. 2). As shown in the heatmap, our gene signature was significantly associated with fustate xpression (Fig. 4c).The correlation between m7G-lncRNA expression and clinicopathological features was then analyzed. In the T stage, SRP14-AS1 exhibited significant differences across groups (*p* < 0.05); in the N stage, MIR22HG showed differences among groups (*p* < 0.001); in the M stage, LINC02178 had differences among groups (*p* < 0.05). MIR22HG was also significantly different between the two groups according to Stage (*p* < 0.001) (Fig. 4d). Overall, the above results showed that, m7G-related lncRNAs are associated with the development of LUSC and may be an effective tool for the clinical management of patients.

### Verification of the prognostic model constructed with m7G-related lncRNAs

**Fig. 5:**
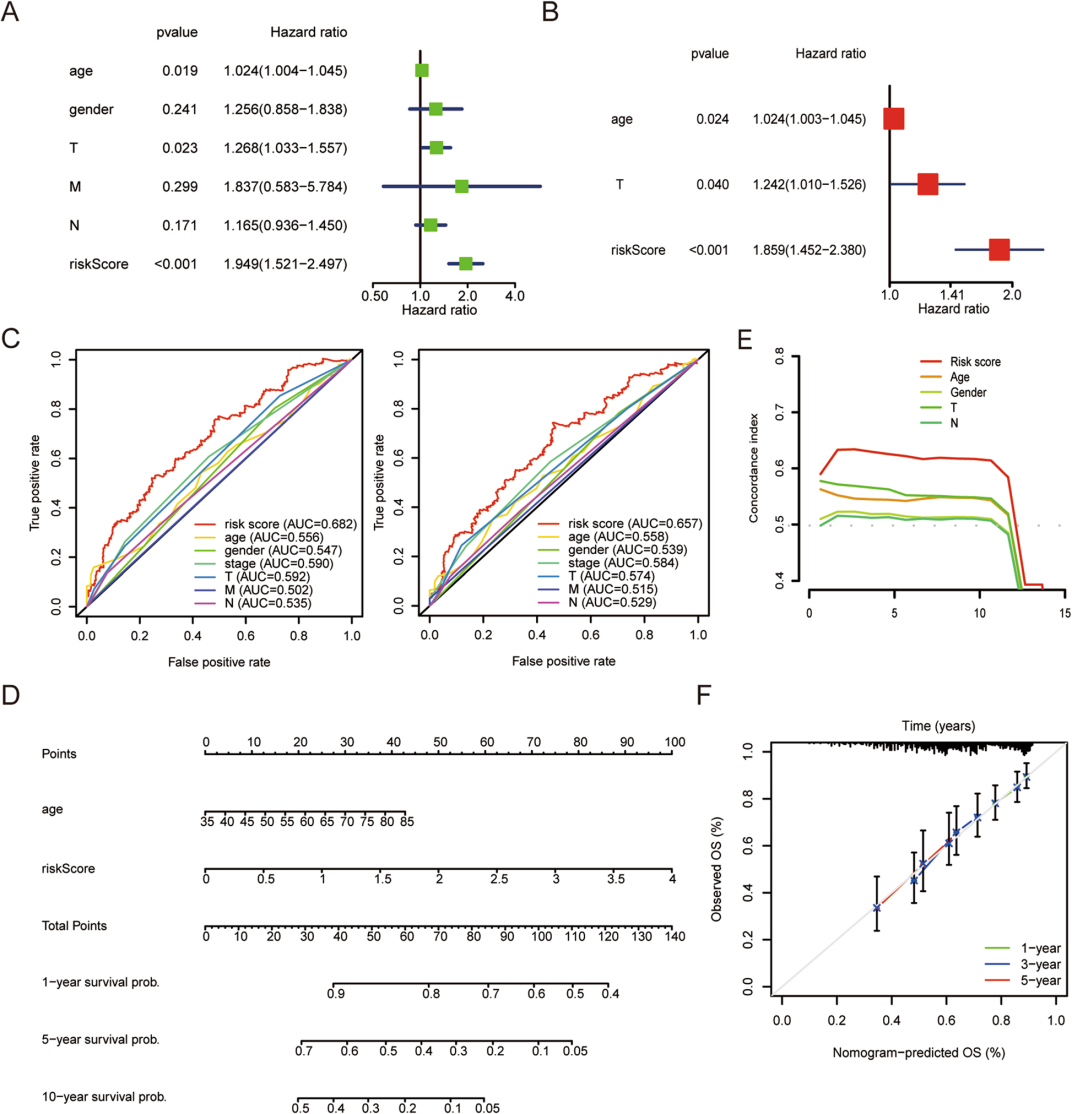
Verification of the risk model. **a**, **b** Univariate and multivariate Cox regression analysis of the prognostic value of risk scores and clinical characteristics. **c** Clinicopathological features and the predictive accuracy of risk models. **d** Construction of the nomogram based on age, T stage and the risk score. **e** C-index of the predictive reliability of the risk model. **f** Calibration curves illustrated agreement between predicted 1-, 3-, and 5-year survival rates and observed outcomes

Univariate and multivariate Cox regression analyses were performed to determine whether the constructed risk model could independently predict prognosis. In univariate Cox regression analysis, age (HR: 1.204, 95% CI: 1.004–1.045, *p* < 0.05), T stage (HR: 1.268, 95% CI: 1.033–1.557, *p* < 0.05), and risk score (HR: 1.949, 95% CI: 1.521–2.497, *p* < 0.001) were significantly associated with OS (Fig. 5a). In multivariate Cox regression analysis, age (HR: 1.024, 95% CI: 1.003–1.045, *p* < 0.05), T stage (HR: 1.242, 95% CI: 1.010–1.526, *p* < 0.05) and risk score (HR: 1.859, 95% CI: 1.452–2.380, *p* < 0.001) were independent prognostic factors in patients with LUSC (Fig. 5b).The area under the ROC curve (AUC) value of the risk score was determined to evaluate its specificity and sensitivity in predicting LUSC prognosis. The AUC values of the risk scores for predicting 3-, and 5-year prognosis were 0.682, and 0.657, respectively, indicating moderate predictive accuracy. These values were higher than those of the other clinicopathological factors (Fig. 5c). Simultaneously, age, T stage and the risk score were used to construct a nomogram to quantitatively predict patient prognosis (Fig. 5d). The C-index of the risk score was higher than that of the other clinical indicators (Fig. 5e). In addition, we used calibration curves to compare the agreement between actual and predicted patient survival at 1, 3, and 5 years. We found that the actual and predicted lines almost agree within 5 years (the closer the line was to 45 degrees or the gray line in the figure, the better the fit) (Fig. 5f). These results indicate that the m7G-related lncRNA risk model can independently predict the prognosis of patients with LUSC.

### PCA verification and GSEA

**Fig. 6:**
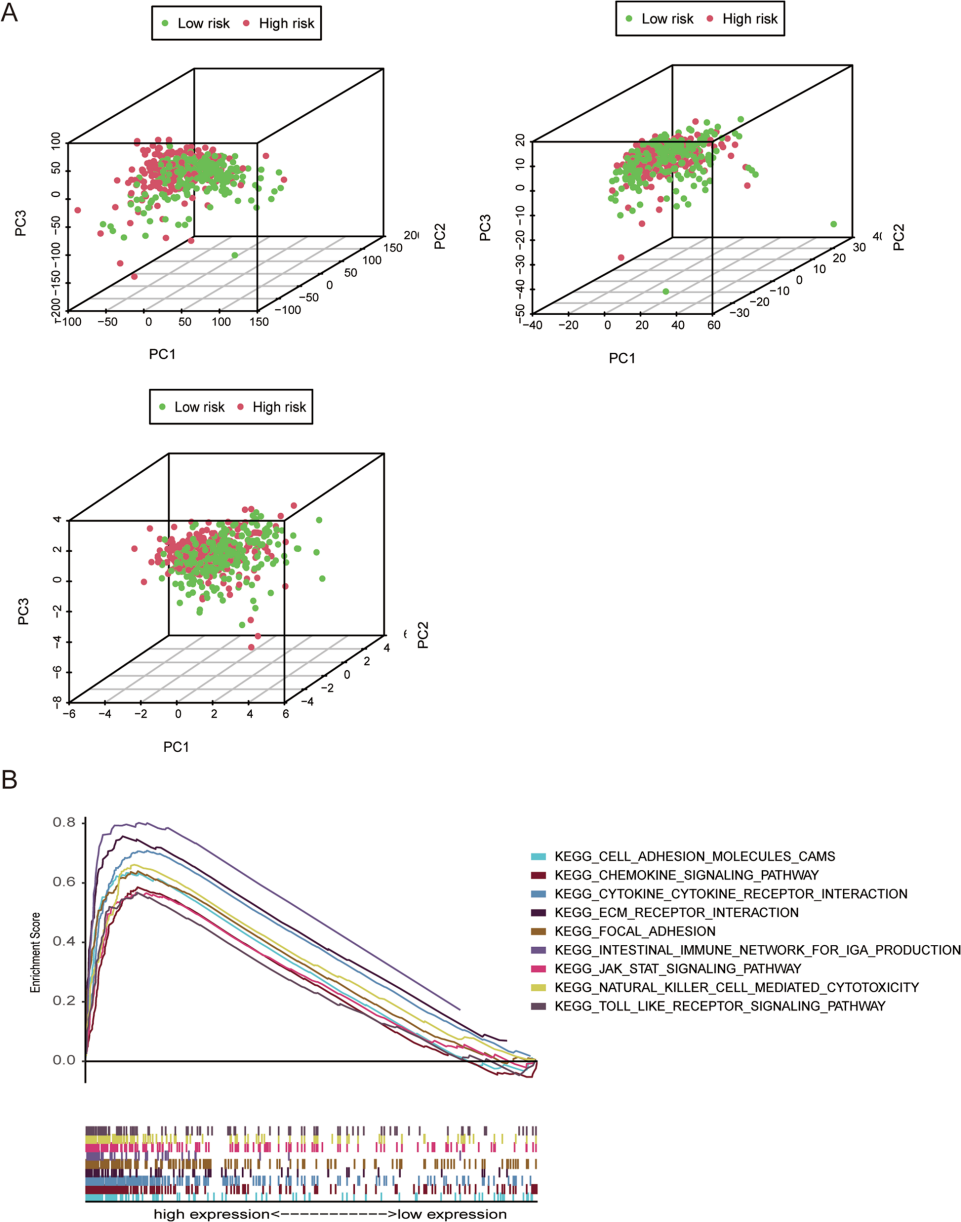
Principal component analysis (PCA) and gene set enrichment analysis (GSEA). **a** All genes, m7G-related lncRNAs and risk genes. **b** Gene set enrichment analysis of m7G-related lncRNAs

PCA was used to evaluate the differences in three expression profiles (total gene expression profiles, m7G-related lncRNAs, and risk model-related lncRNAs) between the low- and high-risk groups. The separation between the high- and low-risk groups was clearer in the risk model related to the lncRNA expression profile than in the other two expression profiles (Fig. 6a).KEGG pathway enrichment analysis to identify the abnormally activated signaling pathways of m7G-related lncRNAs showed that the risk model was significantly enriched in 47 pathways (FDR < 0.05, NES > 2, *p* < 0.05). The results showed that the high expression of m7G-related lncRNAs was related to the toll-like receptor signaling pathway, cell adhesion molecules pathway, chemokine signaling pathway, JAK-STAT signaling pathway, and natural killer cell-mediated cytotoxicity pathway (Fig. 6b).

### Tumor mutational burden

**Fig. 7:**
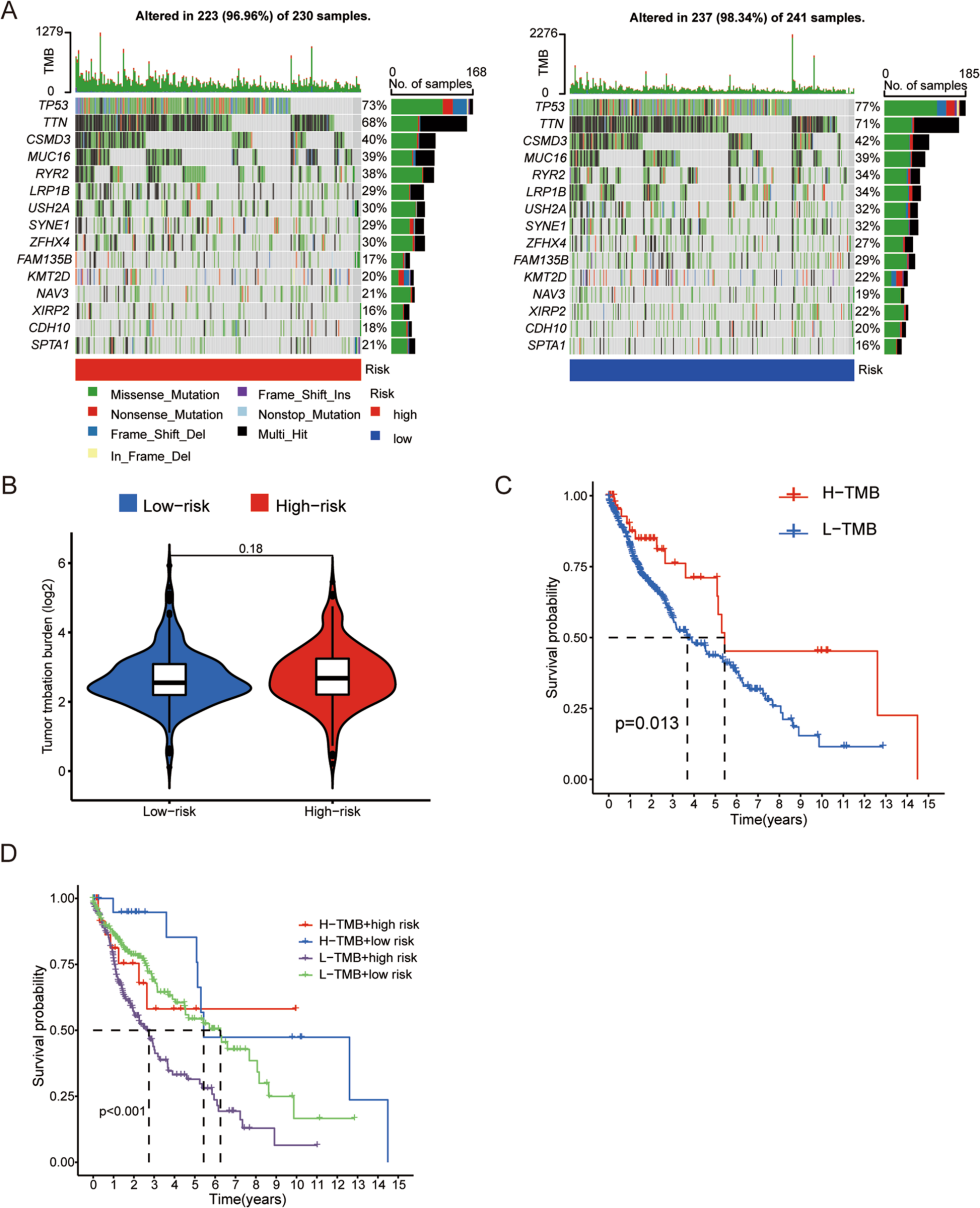
Somatic mutation analysis and tumor mutational burden. **a** Waterfall plot showing the top 15 most frequently mutated genes in the high- and low-risk groups. **b** Differences in TME between the high- and low-risk groups. **c** Kaplan–Meier overall survival (OS) curves in the high-TMB and low-TMB groups. **d** Kaplan–Meier OS curves by TMB groups and risk score groups

TMB represents the gene mutation frequency in the coding region and is associated with tumor progression. Both the high-risk group (96.96%) and the low-risk group (98.34%) had high gene mutation frequencies (Fig. 7a). Particularly, the TP53 gene mutation frequency was the highest (high-risk group: 73%; low-risk group: 77%). However, the TMB was not significantly different between the high- and low-risk groups (Fig. 7b). The samples were divided into the high- and low-mutation groups according to the TMB score, and the high- and low-risk groups were combined for survival analysis. The high tumor burden group (H-TMB) had better prognosis than did the low tumor burden group (L-TMB) (*p* < 0.05) (Fig. 7c). In addition, in L-TMB group, the high-risk group had the worst prognosis than the low-risk group (Fig. 7d).

### Correlation between the risk model and tumor immune microenvironment of LUSC

**Fig. 8:**
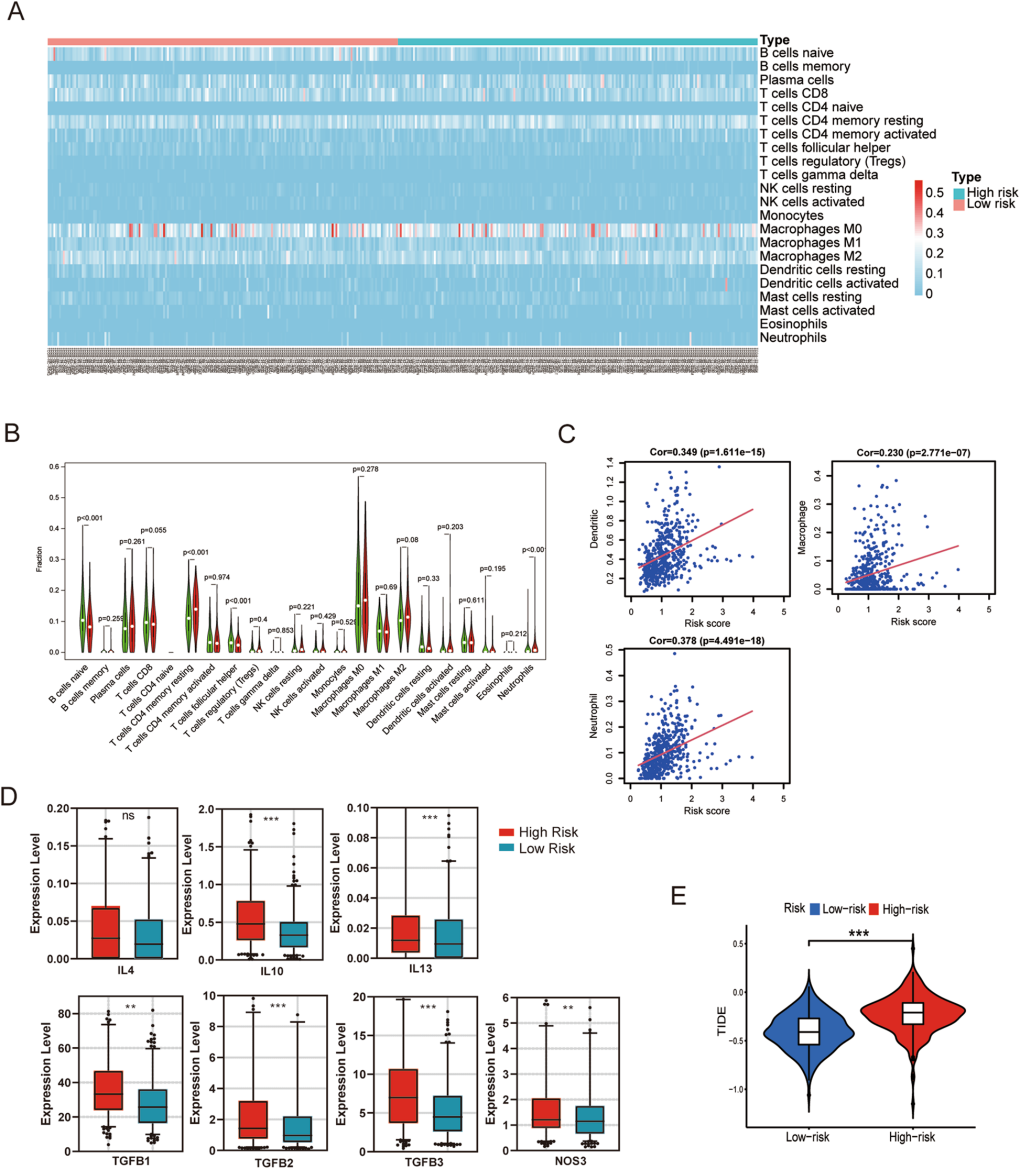
Immune signatures of different risk groups. **a**, **b** Heatmap showing the infiltration levels of 22 immune cells in the high- and low-risk groups. **c** The TIMER database is used to analyze the correlation between the risk score and 6 immune cell subtypes. **d** Expression levels of immunosuppressive cytokines between high and low risk groups. **e** TIDE (Tumor Immune Dysfunction and Exclusion) score between high—and low-risk groups. **p < 0.01, and ***p < 0.001. ns, no sense

Interestingly, GSEA enriched pathways were found to be associated with immune pathways in risk models constructed based on m7G-related lncRNAs. Therefore, we investigated the correlation between the risk model and immune cell function. CIBERSORT was used to screen for *p* values < 0.05. CD4 naive T cells were not expressed in the high- and low-risk groups (Fig. 8a). The expression of resting CD4 memory T cells and neutrophils was higher in the high-risk group than in the low-risk group (*p* < 0.05). In contrast, the levels of naive B cells and follicular helper T cells were lower in the high-risk group than in the low-risk group (*p* < 0.05) (Fig. 8b). Analysis of the correlation among 22 subtypes of immune cells showed that the strongest correlation was between CD4 memory T cells and CD8 T cells (*r* = 0.59), followed by that between CD8 T cells and M0 macrophages (*r* = -0.54) (Supplementary Fig. 3a). The TIMER database was used to analyze the risk scores and six types of immune-infiltrating cells. The results showed that risk score was moderately correlated with dendritic cells, macrophages, and neutrophils (*p* < 0.05), but weakly correlated with B cells, CD4 + T cells, CD8 + T cells (Fig. 8c and Supplementary Fig. 3b). In addition, we explored the relationship between several immune-related molecules and risk score, and the expression levels of immunosuppressive cytokines in the high-risk group were significantly higher compared with levels in the low-risk group (Fig. 8d). In Fig. 8e, the TIDE scores of patients in the low-risk group was lower than that in the high-risk group, suggesting that patients in the low-risk group was more sensitive to immune checkpoint blockade (ICB) therapy. These findings indicate that the risk model constructed based on m7G-related lncRNAs can differentiate the characteristics of tumor immune cells in LUSC.

### Overall survival analysis of six m7G-related lncRNAs and biological function of SRP14-AS1 in vitro

**Fig. 9:**
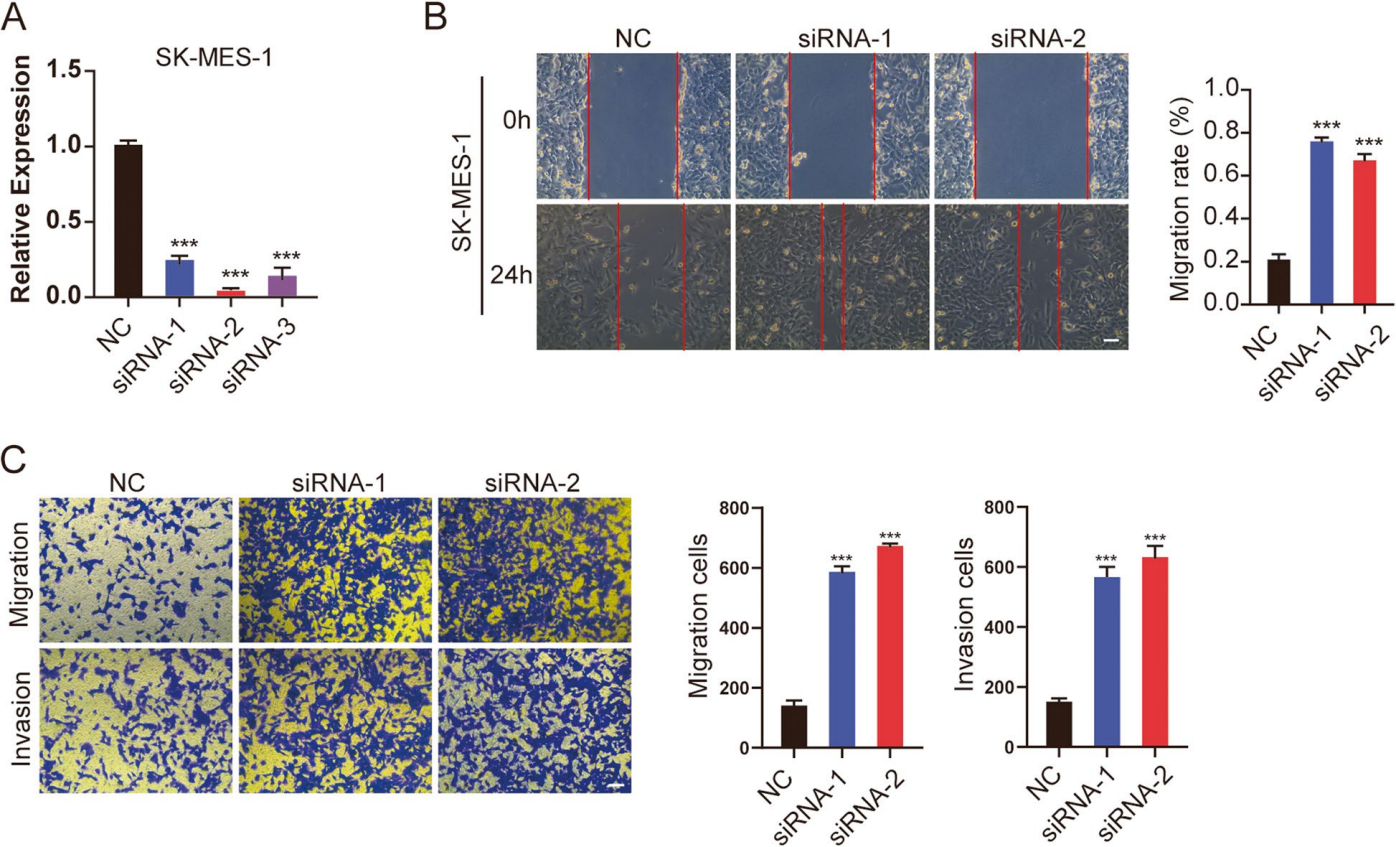
Knockdown of SRP14-AS1 promoted the viability of LUSC cells. **a** SRP14-AS1 knockdown efficiency verification. **b** Representative micrographs of wound-healing experiments. For transfected SK-MES-1 cells, the cell monolayer was scratched with a 10ul sterile pipette tip. Photos were taken at 0 h and 48 h after scratching. **c** Transwell or matrigel-coated transwell was used for cell migration and invasion assays. Scale bars, 100 um. **p < 0.01, and ***p < 0.001

Then, we analyzed the survival of six m7G-related lncRNAs respectively. Among them, CYP4F26P and SRP14-AS1 expression group had prognostic value in overall survival. Moreover, the prognosis of the high SRP14-AS1 expression group was better than that of the low expression group, which was consistent with the results of Fig. 4a that SRP14-AS1 was low expressed in cancer tissues (Supplementary Fig. 4). These results suggested that SRP14-AS1 may function as a tumor suppressor gene. Then, we transfected SK-MES-1 cells with siRNAs, and RT-qPCR showed that siRNA-2, siRNA-3 knockdown efficiency was higher, which was used for subsequent studies (Fig. 9a). A wound-healing assay and transwell migration and invasion assays, indicated that knockdown of SRP14-AS1 accelerated SK-MES-1 cell migration and invasion (Fig. 9b, c). Taken together, the above-presented results suggested that SRP14-AS1 may play a role as a cancer suppressor gene in LUSC.

## Discussion

LUSC is a subtype of NSCLC that accounts for approximately 40% of all lung cancers. Although LUSC is associated with poorer clinical outcomes, it lacks targeted drug agents [25, 26]. Further, basic biomarkers and precise targets for LUSC development and progression remain unknown. Numerous lncRNAs have regulatory functions in the occurrence and development of LUSC [18, 27]. M6A, m5C, m1A, and m7G are common posttranscriptional modifications that play important roles in disease progression [28]. Current studies on the post-transcriptional modification of lncRNAs are mainly focused on m6A and m5C. Meanwhile, m7G-related studies are rare, and its mechanism has not been completely determined. In the current study, m7G-related lncRNAs were divided into subgroups according to their gene expression, and prognostic markers were constructed in combination with survival data. The relationship of the prognostic model based on m7G-related lncRNAs with immune cell infiltration and TMB was then explored. The results showed that the prognostic model based on six m7G-related lncRNAs showed good prognostic value and it has important significance in the evaluation of TMB and tumor immune infiltration. These findings can be used to guide the future clinical diagnosis and treatment of LUSC.

lncRNAs have attracted considerable research attention owing to their key roles in various biological events, such as genome expression and cell differentiation [29, 30]. Abnormal lncRNA expression is also associated with tumorigenesis and tumor progression [31, 32]. Xue et al. found that the m6A methyltransferase METTL3 enhances the stability of the lncRNA ABHD11-AS1 transcript to increase its expression, thereby promoting the Warburg effect in NSCLC, and is closely related to the poor prognosis of patients with NSCLC [33]. In cholangiocarcinoma, the m5C methyltransferase NSUN2 interacts with the lncRNA NKILA to increase its m5C level and promote its interaction with YBX1. In contrast, NKILA interacts with mir-582-3p regulated by m6A methyltransferase METTL3 and inhibits its expression, thereby accelerating cholangiocarcinoma progression through YAP1 [34]. Therefore, RNA methylation is closely related to lncRNA expression, and m7G may affect tumorigenesis by modifying lncRNAs and affecting their stability. However, there are few reports on the modification of the m7G gene in lncRNAs, which may be partly due to the immature detection technology.

The current study analyzed the correlation between 293 m7G-related lncRNAs expression and LUSC prognosis. Six m7G-related lncRNAs were identified to have prognostic value. A prognostic model based on the identified m7G-related lncRNAs was then established. The patients were divided into the high- and low-risk groups based on the median risk score, and the low-risk group was found to have better prognosis than the high-risk group. Further, the risk score could be used as an independent prognostic factor. The AUC value, PCA, and C-index values verified the reliability of the model. Previous studies on the potential of lncRNAs as novel tumor biomarkers have focused on single molecules. However, lncRNAs are not sufficient biomarkers in cancers. To our best knowledge, this is the first study to report an LUSC risk score model based on six m7G-related lncRNAs with prognostic value. Among the six lncRNAs, CYP4F26P has been previously reported to be involved in LUSC. Meanwhile, PTCSC2 [35, 36], LINC02178 [37, 38], SRP14-AS1 [39], and MIR22HG [40, 41] have been mainly reported in other cancer types, such as head and neck squamous cell carcinoma, oral and oropharyngeal squamous cell carcinoma, and thyroid cancer.TMEM99 has not been reported previously.

TMB indicates the total number of somatic mutations that occur in a specific region of the tumor genome. A higher TMB can indirectly reflect the ability of tumors to form more neoantigens, and thus, it may be used as a biomarker for the efficacy of immune checkpoint blockade (ICB) treatment [42, 43]. Previous studies have shown a high TMB in NSCLC [44] and that the TMB level varies greatly according to the smoking status [45]. TMB was higher in LUSC than in other solid tumors [46]. In the current study, both high- and low-risk groups had high somatic mutation frequencies, consistent with previous finding [44].

The H-TMB group had better prognosis than did the L-TMB group, which may be related to the treatment response to ICB drugs in H-TMB patients. GSEA showed that risk profiles are enriched in several immune-related pathways and immune-related diseases, such as the toll-like receptor signaling pathway, natural killer cell-mediated cytotoxicity pathway, and intestinal immune network for IgA production. The current study further analyzed the correlation between the risk model and distribution of tumor-infiltrating immune cells. The results showed that the risk score was positively correlated with dendritic cells, macrophages, and neutrophils (*p* < 0.05), and the risk model could distinguish different characteristics of tumor-infiltrating immune cells in LUSC. To our best knowledge, this study is the first to investigate the relationship between m7G-related lncRNAs and immune cells in LUSC.

However, this study also had some limitations. Although we verified the stability of the risk model using various methods, the model was not externally validated because lncRNA information is lacking in other databases. Further large-scale studies are needed to draw definitive conclusions. Future studies should further explore the functions of these six lncRNAs in LUSC.

In conclusion, we constructed a novel prognostic risk profile consisting of six m7G-related lncRNAs. The risk profile reflected the immune characteristics of patientswith LUSC and showed a high reliability for prognostic prediction. The current study findings provide evidence for further studies on post-transcriptional modifications of lncRNAs and for the development of clinically individualized therapy.

## Supplementary Information

**Additional file 1:** **Supplementary Fig. 1.** Co-expression intensity of prognostic m7G-related IncRNAs and their associated mRNAs.

**Additional file 2:** **Supplementary Fig. 2. **RT-qPCR was used to detect the expression of m7G-related IncRNAs. *p<0.05, **p<0.01, and ***p<0.001.

**Additional file 3:** **Supplementary Fig. 3.** The correlation between risk score and immune cell infiltration was analyzed. **A** Analysis of the correlation among 22 subtyopes of immune cells. **B** The TIMER data base was used to analyze the risk scores and six types of immune-infiltrating cells.

**Additional file 4:** **Supplementary Fig. 4.** Overall survival analysis of six m&G-related IncRNAs in high and low ezpression groups.

**Additional file 5:** **Supplementary Table 1. **The primer sequences involved in this study.

**Additional file 6:** **Supplementary Table 2.** siRNA sequence of SRP14-AS1.

**Additional file 7:** **Supplementary Table 3. **The correlations between lncRNAs and m7G methylated genes.

## Data Availability

All data generated or analysed during this study are included in this published article [and its supplementary information files].

## Abbreviations

C-index: Concordance index
FDR: False discovery rate
GSEA: Gene set enrichment analysis
HCC: Hepatocellular carcinoma
HR: Hazard ratios
ICB: Immune checkpoint blockade
KEGG: Kyoto Encyclopedia of Genes and Genomes
lncRNAs: Long non-coding RNAs
LUAD: Lung adenocarcinoma
LUSC: Lung squamous cell carcinoma
m5C: 5-Methylcytosine
m6A: N6-methyladenosine
m7G: N7-Methylguanosine
METTL1: Methyltransferase-like 1
mRNAs: Messenger RNAs
NSCLC: Non-small cell lung cancer
OS: Overall survival
PCA: Principal component analysis
ROC: Receiver operating characteristic
TCGA: The Cancer Genome Atlas
TMB: Tumor mutational burden
tRNAs: Transfer RNAs
WDR4: WD repeat domain 4
