# Supplementary figures and images for: M7G-Related lncRNAs predict prognosis and regulate the immune microenvironment in lung squamous cell carcinoma
Source: BMC Cancer. 2022 Nov 4;22:1132. doi: 10.1186/s12885-022-10232-z (PMC9636639; doi:10.1186/s12885-022-10232-z)

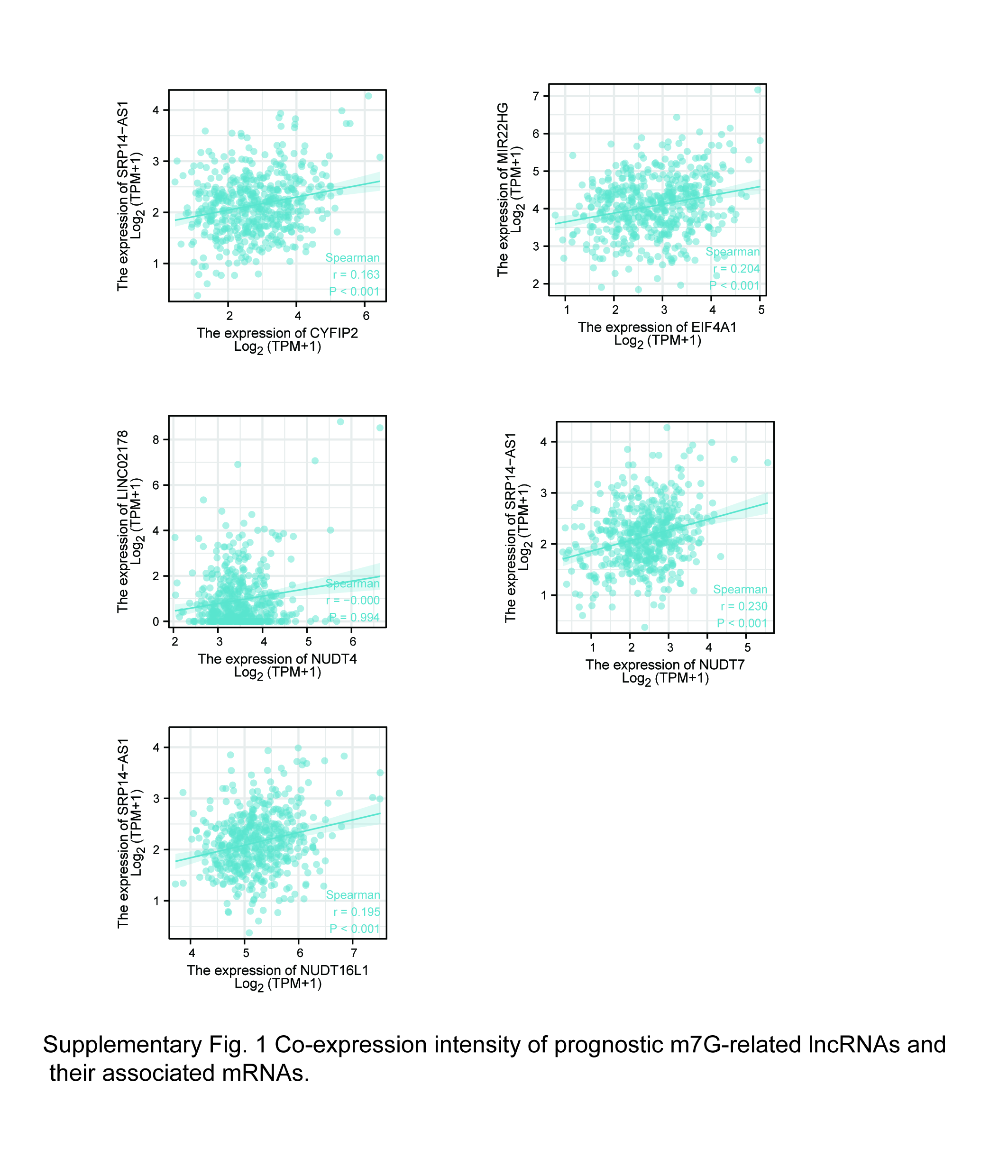

Supplement: Supplementary file 1 — Additional file 1: Supplementary Fig. 1. Co-expression intensity of prognostic m7G-related IncRNAs and their associated mRNAs. [file 12885_2022_10232_MOESM1_ESM.tif]

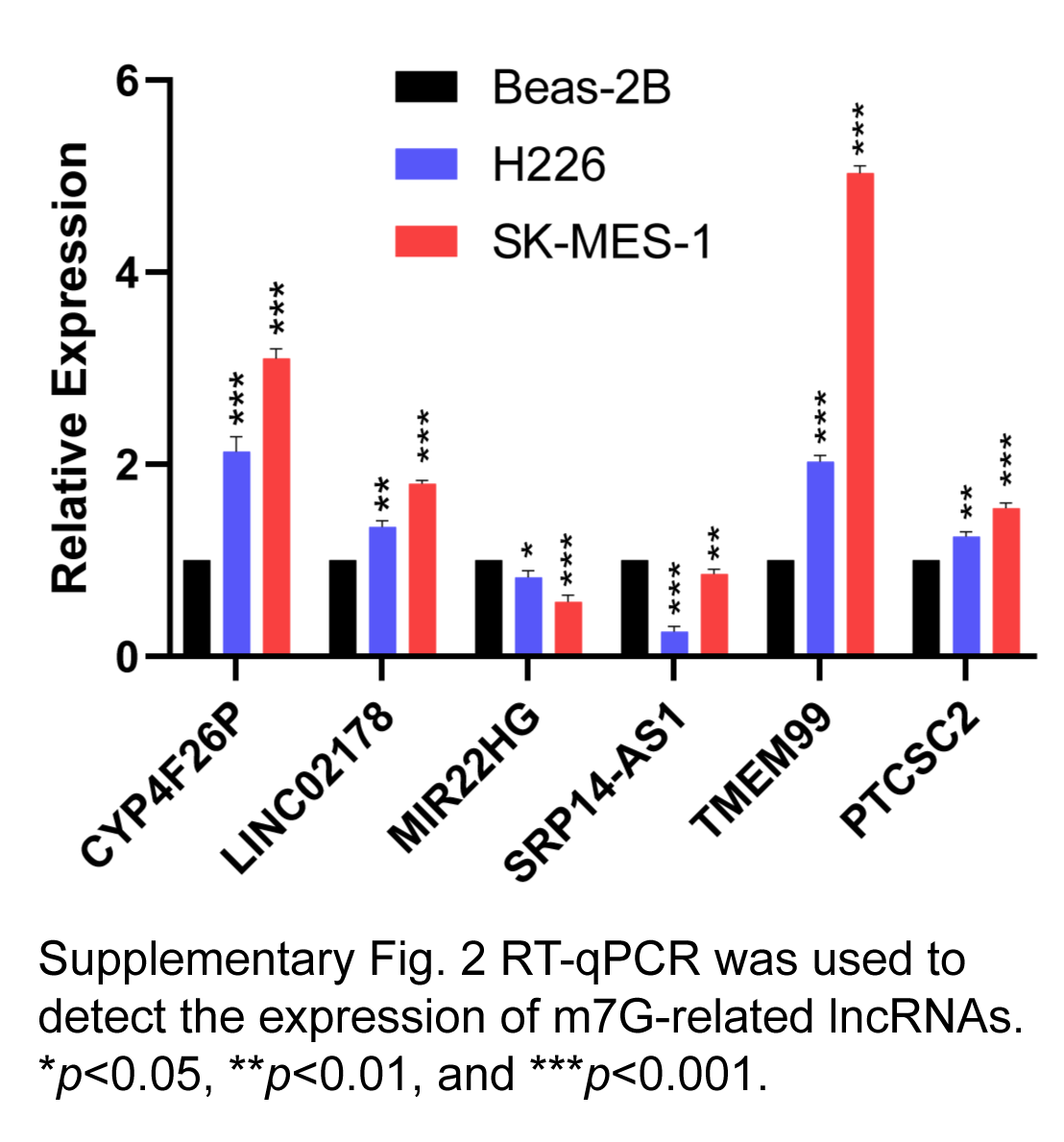

Supplement: Supplementary file 2 — Additional file 2: Supplementary Fig. 2. RT-qPCR was used to detect the expression of m7G-related IncRNAs. *p<0.05, **p<0.01, and ***p<0.001. [file 12885_2022_10232_MOESM2_ESM.tif]

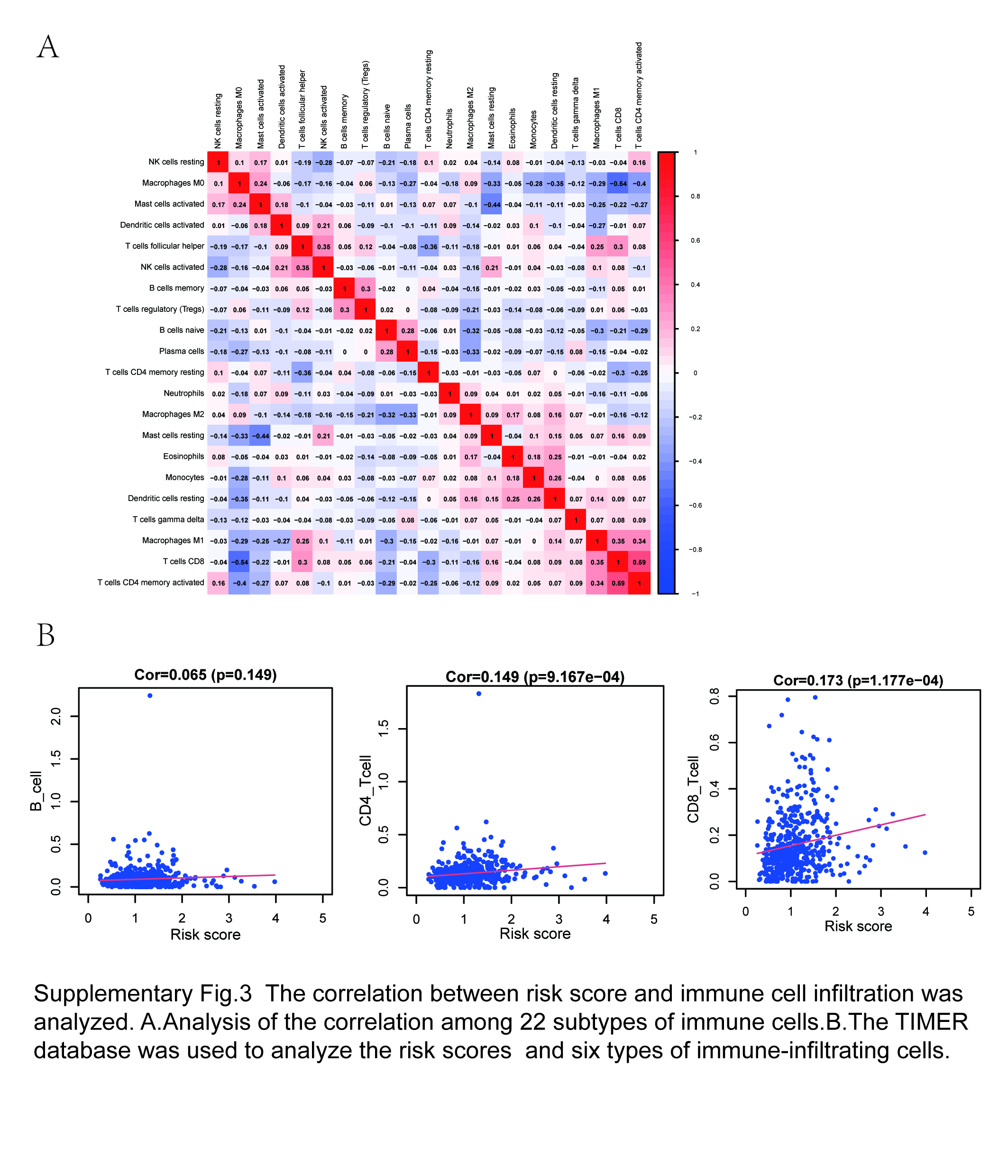

Supplement: Supplementary file 3 — Additional file 3: Supplementary Fig. 3. The correlation between risk score and immune cell infiltration was analyzed. A Analysis of the correlation among 22 subtyopes of immune cells. B The TIMER data base was used to analyze the risk scores and six types of immune-infiltrating cells. [file 12885_2022_10232_MOESM3_ESM.tif]

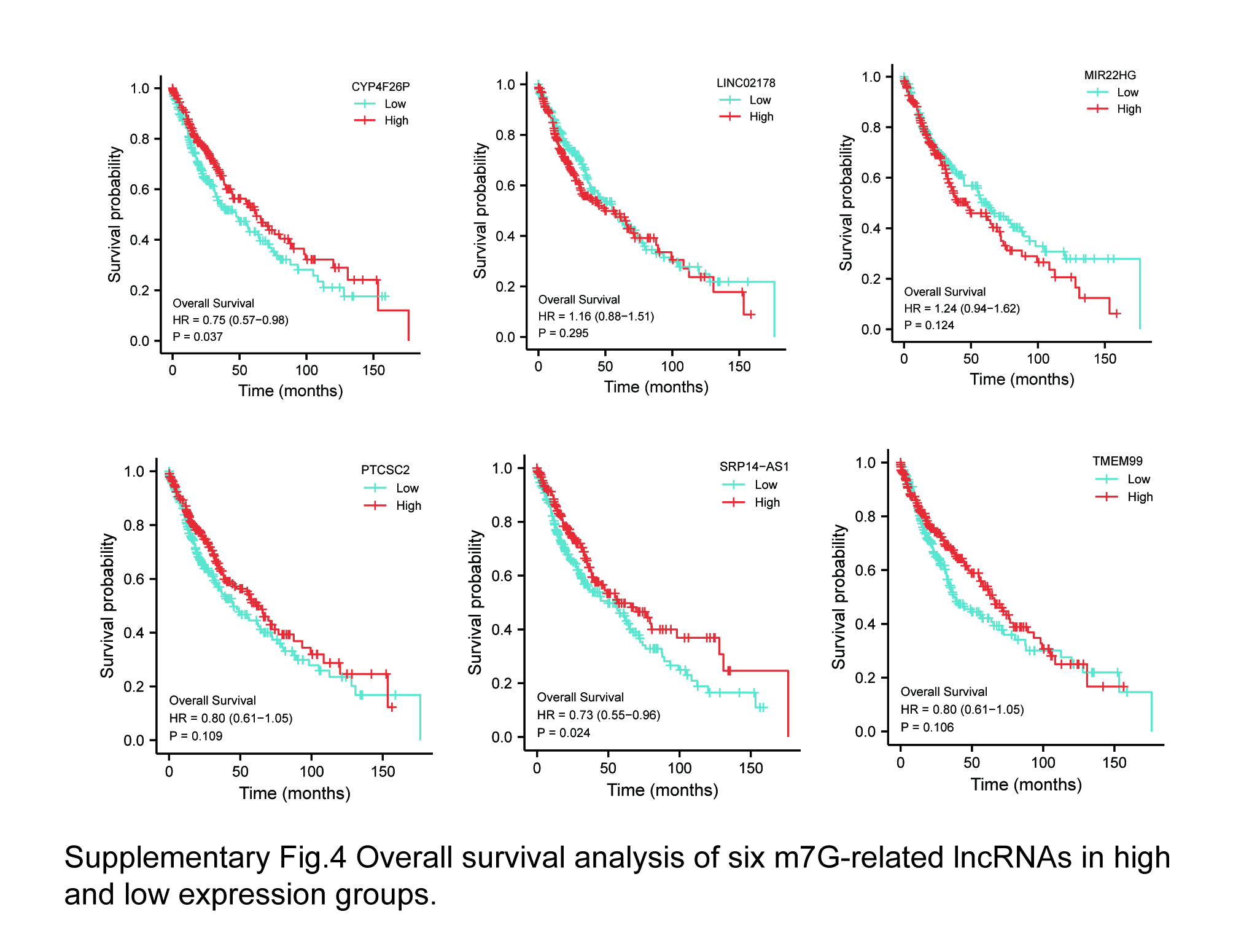

Supplement: Supplementary file 4 — Additional file 4: Supplementary Fig. 4. Overall survival analysis of six m&G-related IncRNAs in high and low ezpression groups. [file 12885_2022_10232_MOESM4_ESM.tif]
