# Supplementary material for: M7G-Related lncRNAs predict prognosis and regulate the immune microenvironment in lung squamous cell carcinoma
Source: BMC Cancer. 2022 Nov 4;22:1132. doi: 10.1186/s12885-022-10232-z (PMC9636639; doi:10.1186/s12885-022-10232-z)
Supplement: Supplementary file 5 — Additional file 5: Supplementary Table 1. The primer sequences involved in this study. [file 12885_2022_10232_MOESM5_ESM.docx]

| Primer | 5' to 3' |
| --- | --- |
| CYP4F26P-F | 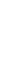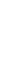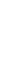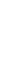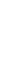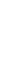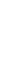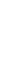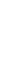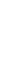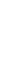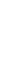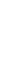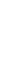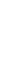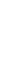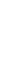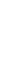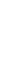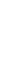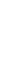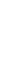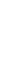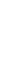CACAGGAGGAGTCACAGAGT |
| CYP4F26P-R | CAGTCTCAGGACCACATTACATC |
| LINC02178-F | 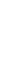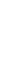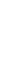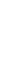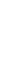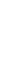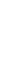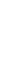CTATGAGTGAGGGTGATGTCAAC |
| LINC02178-R | TCGTGCTGTATGTGCTTCTG |
| MIR22HG-F | TCCAGCCCTCTCCTACTTTG |
| MIR22HG-R | CAGATGGTTCTTGGTCCTTGAG |
| SRP14-AS1-F | TCAAGTGACAGAGGAGCAAGT |
| SRP14-AS1-R | TGGAAGCCGATGAGTTTGTTATG |
| TMEM99-F | TGATAGCCACCACCACCATA |
| TMEM99-R | TGCGAAGTGTCTTAGATGAGTTG |
| PTCSC2-F | TGTGAATGTCCAGGCTTGTT |
| PTCSC2-R | GCTTGCCACAGTATCTTATCAGT |
| GAPDH-F | 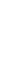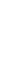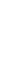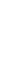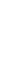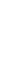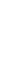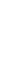GGTGTGAACCATGAGAAGTATGA |
| GAPDH-R | GAGTCCTTCCACGATACCAAAG |

**Supplementary Table 1 The primer sequences involved in this study.**
