# Supplementary material for: M7G-Related lncRNAs predict prognosis and regulate the immune microenvironment in lung squamous cell carcinoma
Source: BMC Cancer. 2022 Nov 4;22:1132. doi: 10.1186/s12885-022-10232-z (PMC9636639; doi:10.1186/s12885-022-10232-z)
Supplement: Supplementary file 6 — Additional file 6: Supplementary Table 2. siRNA sequence of SRP14-AS1. [file 12885_2022_10232_MOESM6_ESM.docx]

| Genes | sense（5'-3'） | antisense（5'-3'） |
| --- | --- | --- |
| hSRP14-AS1 si-1 | GCAGAGAUGGUGUCUUCAAdTdT | UUGAAGACACCAUCUCUGCdTdT |
| hSRP14-AS1 si-2 | GGCUGCAGAGACUAUGUUAdTdT | UAACAUAGUCUCUGCAGCCdTdT |
| hSRP14-AS1 si-3 | GCUGUAUCUCUGAAGGAAAdTdT | UUUCCUUCAGAGAUACAGCdTdT |

**Supplementary Table 2 siRNA sequence of SRP14-AS1.**
