# Supplementary material for: M7G-Related lncRNAs predict prognosis and regulate the immune microenvironment in lung squamous cell carcinoma
Source: BMC Cancer. 2022 Nov 4;22:1132. doi: 10.1186/s12885-022-10232-z (PMC9636639; doi:10.1186/s12885-022-10232-z)
Supplement: Supplementary file 7 — Additional file 7: Supplementary Table 3. The correlations between lncRNAs and m7G methylated genes. [file 12885_2022_10232_MOESM7_ESM.docx]

**Supplementary Table 3 The correlations between lncRNAs and m7G methylated genes.**

| M7Ggene | lncRNA | cor | pvalue |
| --- | --- | --- | --- |
| EIF4A1 | LINC01176 | 0.381869641 | 7.12E-19 |
| NUDT10 | LINC01560 | 0.307552899 | 1.85E-12 |
| CYFIP2 | LINC01857 | 0.53633071 | 9.86E-39 |
| NUDT16L1 | EIF3J-DT | 0.305568487 | 2.61E-12 |
| NCBP3 | EIF3J-DT | 0.300557451 | 6.11E-12 |
| CYFIP2 | EIF3J-DT | 0.320745392 | 1.79E-13 |
| EIF4A1 | MHENCR | 0.382111111 | 6.74E-19 |
| CYFIP2 | MHENCR | 0.368352797 | 1.41E-17 |
| NUDT16L1 | SNHG9 | 0.438115532 | 5.85E-25 |
| TRMT112 | SNHG9 | 0.300577406 | 6.09E-12 |
| EIF4A1 | MYG1-AS1 | 0.519028822 | 5.70E-36 |
| NUDT1 | LINC01063 | 0.379436204 | 1.23E-18 |
| NCBP2 | LINC01063 | 0.450838727 | 1.68E-26 |
| AGO2 | LINC00324 | -0.319472544 | 2.25E-13 |
| DCP2 | HCG27 | 0.374966344 | 3.33E-18 |
| EIF4A1 | HCG27 | 0.341156399 | 3.79E-15 |
| CYFIP2 | HCG27 | 0.418295615 | 1.11E-22 |
| NUDT16L1 | CCNT2-AS1 | 0.338109303 | 6.86E-15 |
| EIF4A1 | CYP4F26P | 0.33744559 | 7.80E-15 |
| NCBP3 | STAG3L5P-PVRIG2P-PILRB | 0.335621526 | 1.11E-14 |
| EIF4A1 | STAG3L5P-PVRIG2P-PILRB | 0.5535473 | 1.21E-41 |
| NUDT7 | SCAMP1-AS1 | 0.317244555 | 3.36E-13 |
| CYFIP2 | LINC00847 | 0.336192973 | 9.93E-15 |
| NSUN2 | C5orf34-AS1 | 0.337039376 | 8.43E-15 |
| NCBP3 | LAMC1-AS1 | 0.341900654 | 3.27E-15 |
| EIF4A1 | LAMC1-AS1 | 0.539258692 | 3.24E-39 |
| NCBP2 | NIFK-AS1 | 0.318015888 | 2.93E-13 |
| DCP2 | RFX5-AS1 | 0.449851565 | 2.22E-26 |
| CYFIP2 | RFX5-AS1 | 0.416649101 | 1.68E-22 |
| NUDT4 | LINC02178 | 0.583818696 | 3.40E-47 |
| NUDT16L1 | LINC00239 | 0.312300415 | 8.09E-13 |
| NUDT7 | TMEM44-AS1 | 0.338054232 | 6.93E-15 |
| NCBP2 | TMEM44-AS1 | 0.587484267 | 6.59E-48 |
| NCBP1 | CIRBP-AS1 | 0.370022997 | 9.81E-18 |
| NCBP3 | CIRBP-AS1 | 0.327669547 | 4.99E-14 |
| DCP2 | LINC00926 | 0.303715467 | 3.58E-12 |
| CYFIP2 | LINC00926 | 0.552403083 | 1.91E-41 |
| NUDT1 | SNHG26 | 0.341658597 | 3.43E-15 |
| EIF4A1 | OCIAD1-AS1 | 0.405478043 | 2.74E-21 |
| NUDT16L1 | MEG3 | 0.314471612 | 5.51E-13 |
| CYFIP2 | MEG3 | 0.326075072 | 6.71E-14 |
| NCBP1 | SBF2-AS1 | 0.313047516 | 7.09E-13 |
| EIF4A1 | LINC00205 | 0.320604741 | 1.83E-13 |
| EIF4G3 | LINC00205 | 0.332776255 | 1.91E-14 |
| SNUPN | UBL7-AS1 | 0.46225547 | 6.08E-28 |
| RAMAC | UBL7-AS1 | 0.505649178 | 6.10E-34 |
| DCP2 | IRF1-AS1 | 0.355685266 | 2.04E-16 |
| EIF4E3 | IRF1-AS1 | 0.415306616 | 2.37E-22 |
| CYFIP2 | IRF1-AS1 | 0.478687973 | 4.10E-30 |
| NUDT1 | LINC01703 | 0.335047042 | 1.24E-14 |
| EIF4A1 | ALG13-AS1 | 0.506020974 | 5.38E-34 |
| EIF4G3 | MRPL20-AS1 | 0.37556809 | 2.91E-18 |
| LSM1 | MRPL20-AS1 | 0.32758915 | 5.07E-14 |
| NUDT16L1 | WASL-DT | 0.35648955 | 1.73E-16 |
| NCBP1 | ARRDC1-AS1 | 0.350607202 | 5.76E-16 |
| NUDT16L1 | LINC02482 | 0.389427557 | 1.26E-19 |
| TRMT112 | LINC02482 | 0.314891058 | 5.12E-13 |
| NUDT16L1 | IDH1-AS1 | 0.309978176 | 1.22E-12 |
| TRMT112 | IDH1-AS1 | 0.310456337 | 1.12E-12 |
| NUDT4 | LINC00920 | 0.364572929 | 3.17E-17 |
| DCP2 | LINC00623 | 0.304484034 | 3.14E-12 |
| CYFIP2 | LINC00623 | 0.310090584 | 1.19E-12 |
| NUDT16L1 | GIHCG | 0.371818374 | 6.64E-18 |
| METTL1 | GIHCG | 0.377856245 | 1.75E-18 |
| TRMT112 | GIHCG | 0.305194266 | 2.78E-12 |
| NCBP1 | FZD10-AS1 | 0.37747529 | 1.91E-18 |
| NUDT1 | DLG5-AS1 | 0.305808167 | 2.50E-12 |
| EIF4A1 | LINC01876 | 0.394324453 | 4.01E-20 |
| AGO2 | CENATAC-DT | 0.304791778 | 2.98E-12 |
| IFIT5 | CCR5AS | 0.410687555 | 7.56E-22 |
| NUDT16 | STARD7-AS1 | 0.340101522 | 4.66E-15 |
| EIF4A1 | LINC00115 | 0.567954335 | 3.25E-44 |
| NUDT16L1 | ZNF503-AS2 | 0.364667309 | 3.10E-17 |
| AGO2 | LINC02449 | 0.302214474 | 4.62E-12 |
| NUDT11 | LINC02542 | 0.307313302 | 1.93E-12 |
| NUDT16L1 | DNAJC9-AS1 | 0.458579404 | 1.79E-27 |
| NUDT7 | DNAJC9-AS1 | 0.36118278 | 6.49E-17 |
| TRMT112 | DNAJC9-AS1 | 0.373847811 | 4.26E-18 |
| NUDT1 | SNHG19 | 0.332924297 | 1.86E-14 |
| EIF4A1 | SNHG10 | 0.304729746 | 3.01E-12 |
| EIF4A1 | LINC00685 | 0.397096841 | 2.08E-20 |
| DCP2 | ADNP-AS1 | 0.335187645 | 1.20E-14 |
| NCBP3 | ADNP-AS1 | 0.302061876 | 4.74E-12 |
| EIF4A1 | ADNP-AS1 | 0.426781784 | 1.22E-23 |
| DCP2 | EPB41L4A-AS1 | 0.303906911 | 3.47E-12 |
| TRMT112 | PRRT3-AS1 | 0.346077235 | 1.43E-15 |
| NUDT7 | DLGAP1-AS1 | 0.315919105 | 4.26E-13 |
| EIF4A1 | PDXDC2P-NPIPB14P | 0.558901785 | 1.39E-42 |
| EIF4E3 | HEIH | 0.383524079 | 4.89E-19 |
| CYFIP2 | HEIH | 0.497816675 | 8.57E-33 |
| NUDT7 | RBPMS-AS1 | 0.320347143 | 1.92E-13 |
| EIF4A1 | MIR22HG | 0.324729208 | 8.61E-14 |
| NUDT1 | ZFAND2A-DT | 0.466129051 | 1.92E-28 |
| NUDT16L1 | ZNF460-AS1 | 0.301785676 | 4.97E-12 |
| EIF4A1 | ZNF460-AS1 | 0.330414518 | 2.98E-14 |
| CYFIP2 | HLA-DQB1-AS1 | 0.374962634 | 3.33E-18 |
| EIF4E3 | USP30-AS1 | 0.364219089 | 3.41E-17 |
| IFIT5 | USP30-AS1 | 0.431643179 | 3.37E-24 |
| CYFIP2 | USP30-AS1 | 0.343729428 | 2.28E-15 |
| EIF4A1 | ATP1B3-AS1 | 0.466629407 | 1.65E-28 |
| NCBP3 | LENG8-AS1 | 0.340622706 | 4.20E-15 |
| EIF4A1 | LENG8-AS1 | 0.501740404 | 2.30E-33 |
| EIF4A1 | SNHG3 | 0.439314524 | 4.21E-25 |
| WDR4 | SNHG3 | 0.311687012 | 9.01E-13 |
| EIF4E3 | FGD5-AS1 | 0.360592164 | 7.35E-17 |
| NCBP3 | LINC00265 | 0.376077267 | 2.60E-18 |
| EIF4A1 | LINC00265 | 0.408159784 | 1.42E-21 |
| EIF4A1 | PVT1 | 0.302979949 | 4.06E-12 |
| EIF4G3 | PINK1-AS | 0.315331182 | 4.73E-13 |
| NUDT1 | SLC12A9-AS1 | 0.316195986 | 4.05E-13 |
| DCP2 | PCED1B-AS1 | 0.305185132 | 2.79E-12 |
| EIF4E3 | PCED1B-AS1 | 0.396058528 | 2.66E-20 |
| CYFIP2 | PCED1B-AS1 | 0.526511039 | 3.81E-37 |
| NUDT16 | LINC02035 | 0.385928329 | 2.82E-19 |
| IFIT5 | PCAT6 | -0.339329168 | 5.41E-15 |
| IFIT5 | PRKAR1B-AS1 | 0.40354485 | 4.40E-21 |
| AGO2 | CYTOR | -0.357017534 | 1.55E-16 |
| EIF4E2 | CYTOR | 0.413967724 | 3.32E-22 |
| DCP2 | ZNF436-AS1 | 0.316628402 | 3.75E-13 |
| NCBP3 | ZNF436-AS1 | 0.319565404 | 2.21E-13 |
| EIF4A1 | ZNF436-AS1 | 0.405217093 | 2.92E-21 |
| EIF4G3 | ZNF436-AS1 | 0.371114057 | 7.74E-18 |
| NUDT16L1 | LINC01023 | 0.312602334 | 7.67E-13 |
| AGO2 | NCBP2-AS1 | 0.356882652 | 1.59E-16 |
| NCBP2 | NCBP2-AS1 | 0.482813886 | 1.12E-30 |
| NCBP3 | NCBP2-AS1 | 0.319337364 | 2.30E-13 |
| EIF4A1 | NCBP2-AS1 | 0.587133001 | 7.72E-48 |
| NUDT16 | LPP-AS2 | 0.317218876 | 3.38E-13 |
| NCBP2 | LPP-AS2 | 0.4586819 | 1.74E-27 |
| IFIT5 | HCP5 | 0.47437614 | 1.56E-29 |
| NCBP2 | TTC28-AS1 | 0.30460445 | 3.08E-12 |
| NUDT16L1 | MAPKAPK5-AS1 | 0.479186458 | 3.51E-30 |
| SNUPN | MAPKAPK5-AS1 | 0.323628738 | 1.05E-13 |
| TRMT112 | MAPKAPK5-AS1 | 0.376764945 | 2.23E-18 |
| RAMAC | MAPKAPK5-AS1 | 0.35043371 | 5.97E-16 |
| NUDT1 | UBAC2-AS1 | 0.342849478 | 2.72E-15 |
| NCBP3 | LINC00106 | 0.383833544 | 4.56E-19 |
| EIF4A1 | LINC00106 | 0.53256527 | 4.06E-38 |
| EIF4E | SNHG8 | 0.375486828 | 2.97E-18 |
| DCP2 | LINC02576 | 0.32843277 | 4.33E-14 |
| CYFIP2 | LINC02576 | 0.455538519 | 4.35E-27 |
| EIF4A1 | LINC00653 | 0.329411243 | 3.60E-14 |
| RNMT | ACVR2B-AS1 | 0.306556619 | 2.20E-12 |
| EIF4A1 | MIR503HG | 0.46351598 | 4.18E-28 |
| EIF4E3 | LINC02362 | 0.334186239 | 1.46E-14 |
| NCBP3 | INE1 | 0.402217454 | 6.07E-21 |
| EIF4A1 | INE1 | 0.635045218 | 4.90E-58 |
| NCBP1 | PPP1R26-AS1 | 0.311920802 | 8.65E-13 |
| NCBP3 | PPP1R26-AS1 | 0.302680714 | 4.27E-12 |
| EIF4A1 | ZNF213-AS1 | 0.382765796 | 5.81E-19 |
| CYFIP2 | ZNF213-AS1 | 0.316700203 | 3.70E-13 |
| EIF4E3 | LINC00582 | 0.321904185 | 1.45E-13 |
| NUDT1 | SNHG1 | 0.315208531 | 4.83E-13 |
| EIF4A1 | SNHG1 | 0.433361026 | 2.12E-24 |
| TRMT112 | SNHG1 | 0.313262869 | 6.83E-13 |
| EIF4A1 | SUGT1-DT | 0.321216694 | 1.64E-13 |
| EIF4E | SNHG16 | 0.316103895 | 4.12E-13 |
| EIF4A1 | MIR222HG | 0.598258485 | 4.70E-50 |
| NCBP1 | SLC25A25-AS1 | 0.369215877 | 1.17E-17 |
| EIF4A1 | SLC25A25-AS1 | 0.41790209 | 1.22E-22 |
| CYFIP2 | LINC01547 | 0.314068185 | 5.92E-13 |
| NSUN2 | PP7080 | 0.440143471 | 3.36E-25 |
| EIF4A1 | CD27-AS1 | 0.342595045 | 2.86E-15 |
| NUDT16L1 | STX17-AS1 | 0.336131395 | 1.00E-14 |
| NUDT7 | STX17-AS1 | 0.363464992 | 4.01E-17 |
| NCBP2 | KCNMB2-AS1 | 0.454504805 | 5.86E-27 |
| NUDT16L1 | MYLK-AS1 | 0.419578644 | 7.96E-23 |
| EIF4A1 | THUMPD3-AS1 | 0.421318089 | 5.09E-23 |
| EIF4G3 | NORAD | 0.30576625 | 2.52E-12 |
| EIF4E3 | LINC01781 | 0.312795761 | 7.41E-13 |
| CYFIP2 | LINC01781 | 0.483913181 | 7.90E-31 |
| AGO2 | MIR4435-2HG | -0.302721587 | 4.24E-12 |
| NCBP3 | PSMA3-AS1 | 0.437464037 | 6.99E-25 |
| EIF4A1 | PSMA3-AS1 | 0.623713298 | 1.83E-55 |
| NUDT16L1 | KMT2E-AS1 | 0.345062247 | 1.75E-15 |
| DCP2 | LINC00641 | 0.352855519 | 3.65E-16 |
| NCBP3 | LINC00641 | 0.376237096 | 2.51E-18 |
| EIF4A1 | LINC00641 | 0.513227436 | 4.44E-35 |
| CYFIP2 | LINC00641 | 0.340010835 | 4.74E-15 |
| NUDT3 | SNHG32 | 0.377351458 | 1.96E-18 |
| NUDT1 | SNHG25 | 0.43539248 | 1.23E-24 |
| NUDT1 | PITPNA-AS1 | 0.306469501 | 2.23E-12 |
| NCBP3 | PITPNA-AS1 | 0.419845987 | 7.44E-23 |
| TRMT112 | PITPNA-AS1 | 0.322029872 | 1.41E-13 |
| NUDT1 | G2E3-AS1 | 0.30580994 | 2.50E-12 |
| NUDT16L1 | LINC01003 | 0.302663503 | 4.28E-12 |
| NUDT16L1 | NEBL-AS1 | 0.367044727 | 1.87E-17 |
| EIF4E2 | GAPLINC | 0.306855818 | 2.09E-12 |
| NUDT1 | TYMSOS | 0.319905208 | 2.08E-13 |
| EIF4A1 | NALT1 | 0.332833468 | 1.89E-14 |
| NCBP3 | SDCBP2-AS1 | 0.300426533 | 6.25E-12 |
| EIF4A1 | SDCBP2-AS1 | 0.330679195 | 2.84E-14 |
| METTL1 | HHIP-AS1 | 0.380660284 | 9.35E-19 |
| EIF4E3 | LINC02489 | 0.364620965 | 3.13E-17 |
| NUDT16L1 | TAF1A-AS1 | 0.348777516 | 8.34E-16 |
| SNUPN | TAF1A-AS1 | 0.341446372 | 3.58E-15 |
| TRMT112 | TAF1A-AS1 | 0.300199679 | 6.49E-12 |
| RAMAC | TAF1A-AS1 | 0.424348563 | 2.32E-23 |
| TRMT112 | FLJ20021 | 0.307339827 | 1.92E-12 |
| DCP2 | SOS1-IT1 | 0.436848314 | 8.27E-25 |
| NUDT7 | NRSN2-AS1 | 0.340678153 | 4.16E-15 |
| EIF4A1 | CD44-AS1 | 0.46618573 | 1.89E-28 |
| EIF4E3 | DTNB-AS1 | 0.328952473 | 3.93E-14 |
| NUDT16L1 | LINC00526 | 0.354474095 | 2.62E-16 |
| RNMT | LINC00526 | 0.348988461 | 7.99E-16 |
| DCPS | GSEC | 0.386763438 | 2.33E-19 |
| NUDT16 | ARHGAP31-AS1 | 0.327075608 | 5.57E-14 |
| RNMT | ARHGAP31-AS1 | 0.319256767 | 2.34E-13 |
| NUDT4 | HAS2-AS1 | 0.314565385 | 5.42E-13 |
| DCP2 | TMEM161B-AS1 | 0.359961828 | 8.38E-17 |
| CYFIP2 | TMEM161B-AS1 | 0.339075505 | 5.69E-15 |
| NUDT1 | POLR2J4 | 0.388337777 | 1.62E-19 |
| NCBP3 | LINC01089 | 0.337355096 | 7.94E-15 |
| EIF4A1 | LINC01089 | 0.444557628 | 9.87E-26 |
| CYFIP2 | LINC01089 | 0.31704342 | 3.48E-13 |
| DCP2 | PTOV1-AS1 | 0.369383965 | 1.13E-17 |
| NUDT16 | BAIAP2-DT | 0.432463549 | 2.70E-24 |
| NUDT16L1 | ELOA-AS1 | 0.308779426 | 1.50E-12 |
| EIF4A1 | MMP25-AS1 | 0.442421455 | 1.79E-25 |
| DCP2 | LINC01138 | 0.384335194 | 4.07E-19 |
| EIF4A1 | LMNTD2-AS1 | 0.35656515 | 1.70E-16 |
| EIF4E3 | HECW2-AS1 | 0.301277126 | 5.42E-12 |
| NCBP3 | RUSC1-AS1 | 0.320355937 | 1.92E-13 |
| EIF4A1 | RUSC1-AS1 | 0.601106497 | 1.23E-50 |
| NCBP3 | SCAT2 | 0.334803196 | 1.30E-14 |
| EIF4A1 | SCAT2 | 0.527585098 | 2.57E-37 |
| EIF4E3 | TBX5-AS1 | 0.319348685 | 2.30E-13 |
| NCBP2 | SNHG20 | 0.301940785 | 4.84E-12 |
| NCBP3 | SNHG20 | 0.307035005 | 2.03E-12 |
| EIF4A1 | SNHG20 | 0.475774556 | 1.01E-29 |
| EIF4E3 | MAGI2-AS3 | 0.383631427 | 4.77E-19 |
| DCP2 | H1-10-AS1 | 0.316731246 | 3.68E-13 |
| NUDT16 | H1-10-AS1 | 0.415850726 | 2.06E-22 |
| EIF4A1 | H1-10-AS1 | 0.371102395 | 7.76E-18 |
| CYFIP2 | H1-10-AS1 | 0.316299797 | 3.98E-13 |
| RAMAC | LINC01806 | 0.318601203 | 2.63E-13 |
| AGO2 | LINC01943 | -0.315405955 | 4.67E-13 |
| EIF4E3 | LINC01943 | 0.325523548 | 7.43E-14 |
| EIF4A1 | LINC01311 | 0.322675275 | 1.26E-13 |
| NCBP2 | LINC02041 | 0.301535848 | 5.18E-12 |
| DCP2 | ZNF32-AS2 | 0.312457737 | 7.87E-13 |
| NCBP3 | ZNF32-AS2 | 0.325805761 | 7.06E-14 |
| EIF4A1 | ZNF32-AS2 | 0.526480706 | 3.85E-37 |
| NUDT3 | RNASEH1-AS1 | 0.315331462 | 4.73E-13 |
| NUDT4 | RNASEH1-AS1 | 0.302731659 | 4.23E-12 |
| NUDT7 | RNASEH1-AS1 | 0.359885054 | 8.52E-17 |
| NCBP2 | SOX21-AS1 | 0.317721557 | 3.08E-13 |
| TRMT112 | PPP1R14B-AS1 | 0.356891212 | 1.59E-16 |
| CYFIP2 | PPP1R14B-AS1 | -0.301260356 | 5.43E-12 |
| EIF4A1 | YEATS2-AS1 | 0.360513357 | 7.47E-17 |
| NUDT1 | RNF216P1 | 0.535070366 | 1.59E-38 |
| NCBP2 | TIPARP-AS1 | 0.360118773 | 8.11E-17 |
| NUDT16L1 | MINCR | 0.31364096 | 6.39E-13 |
| WDR4 | MINCR | 0.327821111 | 4.85E-14 |
| TRMT112 | MINCR | 0.300353437 | 6.33E-12 |
| CYFIP2 | FAM111A-DT | 0.309931285 | 1.23E-12 |
| EIF4E3 | LINC01936 | 0.322661406 | 1.26E-13 |
| EIF4A1 | DTX2P1-UPK3BP1-PMS2P11 | 0.366534918 | 2.08E-17 |
| EIF4A1 | LINC00342 | 0.429141914 | 6.56E-24 |
| LSM1 | LINC02762 | 0.414100296 | 3.21E-22 |
| NUDT16L1 | LOH12CR2 | 0.353563923 | 3.16E-16 |
| NUDT7 | LOH12CR2 | 0.304526382 | 3.12E-12 |
| NCBP3 | RAD51-AS1 | 0.404882617 | 3.17E-21 |
| EIF4A1 | RAD51-AS1 | 0.525289251 | 5.96E-37 |
| CYFIP2 | RAD51-AS1 | 0.322993739 | 1.18E-13 |
| NUDT16L1 | NRAV | 0.34919772 | 7.66E-16 |
| EIF4E2 | NRAV | 0.375352037 | 3.05E-18 |
| TRMT112 | NRAV | 0.333085123 | 1.80E-14 |
| EIF4G3 | MRPL20-DT | 0.303463554 | 3.74E-12 |
| LSM1 | MRPL20-DT | 0.310441261 | 1.12E-12 |
| NUDT1 | TMPO-AS1 | 0.333770547 | 1.58E-14 |
| NCBP1 | TMPO-AS1 | 0.316361303 | 3.94E-13 |
| LARP1 | ASB16-AS1 | 0.303370991 | 3.80E-12 |
| EIF4A1 | ASB16-AS1 | 0.327958006 | 4.73E-14 |
| NSUN2 | LINC01194 | 0.337961709 | 7.06E-15 |
| EIF4A1 | MALAT1 | 0.521560658 | 2.30E-36 |
| NUDT16 | ARHGAP27P1-BPTFP1-KPNA2P3 | 0.307613656 | 1.83E-12 |
| EIF4A1 | ARHGAP27P1-BPTFP1-KPNA2P3 | 0.475749376 | 1.02E-29 |
| NCBP3 | ZKSCAN2-DT | 0.350543371 | 5.84E-16 |
| EIF4A1 | ZKSCAN2-DT | 0.47789316 | 5.26E-30 |
| DCP2 | LINC01963 | 0.304363247 | 3.21E-12 |
| CYFIP2 | LINC01963 | 0.406580232 | 2.09E-21 |
| NCBP2 | SNHG21 | 0.305802708 | 2.51E-12 |
| SNUPN | SNHG21 | 0.343914745 | 2.20E-15 |
| RAMAC | SNHG21 | 0.417626534 | 1.31E-22 |
| DCP2 | SH3BP5-AS1 | 0.379402575 | 1.24E-18 |
| NCBP3 | SH3BP5-AS1 | 0.373911361 | 4.20E-18 |
| EIF4A1 | SH3BP5-AS1 | 0.589547699 | 2.60E-48 |
| CYFIP2 | SH3BP5-AS1 | 0.425077184 | 1.91E-23 |
| NUDT1 | GS1-124K5.4 | 0.335173928 | 1.21E-14 |
| EIF4A1 | ZNRD2-AS1 | 0.313663439 | 6.36E-13 |
| DCP2 | PTOV1-AS2 | 0.34334301 | 2.46E-15 |
| NCBP3 | PTOV1-AS2 | 0.34854002 | 8.75E-16 |
| EIF4A1 | PTOV1-AS2 | 0.668636359 | 2.47E-66 |
| CYFIP2 | UST-AS2 | 0.31116091 | 9.88E-13 |
| NCBP3 | LINC00173 | 0.304447499 | 3.16E-12 |
| EIF4A1 | LINC00173 | 0.398500689 | 1.49E-20 |
| NUDT16L1 | OSER1-DT | 0.446018319 | 6.56E-26 |
| NUDT7 | OSER1-DT | 0.362273095 | 5.16E-17 |
| EIF4A1 | LINC00174 | 0.330559611 | 2.90E-14 |
| EIF4A1 | NEAT1 | 0.681518782 | 8.21E-70 |
| EIF4A1 | ADIRF-AS1 | 0.362668774 | 4.74E-17 |
| NUDT5 | SNHG6 | 0.342816991 | 2.73E-15 |
| AGO2 | LINC01094 | -0.331758426 | 2.31E-14 |
| EIF4E3 | LINC01094 | 0.320611036 | 1.83E-13 |
| IFIT5 | LINC01094 | 0.437813423 | 6.36E-25 |
| EIF4A1 | LINC01004 | 0.469608208 | 6.72E-29 |
| EIF4A1 | THAP9-AS1 | 0.348189483 | 9.39E-16 |
| NUDT1 | ASH1L-AS1 | 0.311132813 | 9.93E-13 |
| DCP2 | ANKRD10-IT1 | 0.358469434 | 1.15E-16 |
| NCBP3 | ANKRD10-IT1 | 0.351530485 | 4.78E-16 |
| EIF4A1 | ANKRD10-IT1 | 0.487742811 | 2.32E-31 |
| NUDT16 | EIF2AK3-DT | 0.348173164 | 9.42E-16 |
| NUDT7 | EIF2AK3-DT | 0.374526003 | 3.67E-18 |
| NUDT16L1 | FLJ37453 | 0.342214648 | 3.08E-15 |
| METTL1 | LINC01436 | 0.533057677 | 3.38E-38 |
| NUDT16L1 | SPINT1-AS1 | 0.309768274 | 1.26E-12 |
| SNUPN | SPINT1-AS1 | 0.390833161 | 9.09E-20 |
| RAMAC | SPINT1-AS1 | 0.366364271 | 2.16E-17 |
| RNMT | LINC01521 | 0.328475438 | 4.29E-14 |
| DCP2 | LINC01355 | 0.384558166 | 3.86E-19 |
| NCBP3 | LINC01355 | 0.347925441 | 9.90E-16 |
| EIF4A1 | LINC01355 | 0.552417536 | 1.90E-41 |
| EIF4A1 | THBS3-AS1 | 0.483645696 | 8.60E-31 |
| EIF4A1 | GARS1-DT | 0.514774978 | 2.58E-35 |
| TRMT112 | ZNF687-AS1 | 0.331274224 | 2.54E-14 |
| EIF4E3 | CARD8-AS1 | 0.37552899 | 2.94E-18 |
| IFIT5 | CARD8-AS1 | 0.36786569 | 1.56E-17 |
| LSM1 | PRANCR | 0.487747762 | 2.32E-31 |
| EIF4E | UBA6-AS1 | 0.3284086 | 4.35E-14 |
| NUDT1 | DGUOK-AS1 | 0.34454464 | 1.94E-15 |
| NCBP3 | GABPB1-AS1 | 0.321335177 | 1.60E-13 |
| EIF4A1 | GABPB1-AS1 | 0.411486575 | 6.19E-22 |
| EIF4A1 | NR4A1AS | 0.329082382 | 3.83E-14 |
| NUDT3 | FOXP4-AS1 | 0.444627812 | 9.68E-26 |
| NCBP3 | ASMTL-AS1 | 0.356668164 | 1.66E-16 |
| EIF4A1 | ASMTL-AS1 | 0.541301309 | 1.48E-39 |
| CYFIP2 | PAXIP1-AS2 | 0.342430931 | 2.95E-15 |
| NCBP3 | BACE1-AS | 0.334167591 | 1.46E-14 |
| EIF4A1 | BACE1-AS | 0.325488036 | 7.48E-14 |
| EIF4A1 | TMEM147-AS1 | 0.367589701 | 1.66E-17 |
| NCBP2 | LINC00885 | 0.453207534 | 8.52E-27 |
| EIF4A1 | NDUFV2-AS1 | 0.397888318 | 1.72E-20 |
| EIF4A1 | MED8-AS1 | 0.556477015 | 3.71E-42 |
| NCBP3 | AGBL5-IT1 | 0.328539816 | 4.24E-14 |
| EIF4A1 | AGBL5-IT1 | 0.484587893 | 6.37E-31 |
| EIF4A1 | PRKCZ-AS1 | 0.396931888 | 2.16E-20 |
| NUDT16 | ERVK13-1 | 0.300722975 | 5.95E-12 |
| NCBP3 | ERVK13-1 | 0.306874884 | 2.08E-12 |
| EIF4A1 | ERVK13-1 | 0.544188931 | 4.85E-40 |
| NUDT11 | LINC02820 | 0.307121089 | 2.00E-12 |
| NUDT16L1 | PXN-AS1 | 0.512114103 | 6.55E-35 |
| EIF4E | PXN-AS1 | 0.309882118 | 1.24E-12 |
| SNUPN | PXN-AS1 | 0.371997004 | 6.39E-18 |
| TRMT112 | PXN-AS1 | 0.426633753 | 1.27E-23 |
| DCP2 | ZNF710-AS1 | 0.3945309 | 3.82E-20 |
| CYFIP2 | ZNF710-AS1 | 0.360611858 | 7.32E-17 |
| NUDT16 | SEMA3B-AS1 | 0.318684385 | 2.59E-13 |
| EIF4A1 | SEMA3B-AS1 | 0.340653678 | 4.18E-15 |
| NCBP2 | MELTF-AS1 | 0.501608479 | 2.41E-33 |
| EIF4A1 | MELTF-AS1 | 0.320669929 | 1.81E-13 |
| LARP1 | SNHG4 | 0.335259852 | 1.19E-14 |
| EIF4A1 | SNHG4 | 0.397448105 | 1.91E-20 |
| EIF4A1 | TNFRSF14-AS1 | 0.352270852 | 4.11E-16 |
| CYFIP2 | TNFRSF14-AS1 | 0.432052566 | 3.02E-24 |
| RNMT | LINC00667 | 0.473873918 | 1.82E-29 |
| NUDT16L1 | MIR924HG | 0.302087219 | 4.72E-12 |
| CYFIP2 | LINC00957 | 0.388622749 | 1.52E-19 |
| NUDT1 | LINC01980 | 0.347553219 | 1.07E-15 |
| NUDT11 | LINC01980 | 0.318651088 | 2.61E-13 |
| EIF4G3 | LINC01128 | 0.39740294 | 1.93E-20 |
| NCBP3 | HM13-IT1 | 0.321885294 | 1.45E-13 |
| EIF4A1 | HM13-IT1 | 0.470650553 | 4.89E-29 |
| NCBP3 | LINC02693 | 0.310233182 | 1.16E-12 |
| RNMT | LINC02693 | 0.309143508 | 1.41E-12 |
| NUDT1 | DCST1-AS1 | 0.340534434 | 4.28E-15 |
| NSUN2 | IRX4-AS1 | 0.338658359 | 6.17E-15 |
| NUDT16L1 | ZSCAN16-AS1 | 0.449846348 | 2.22E-26 |
| NUDT3 | ZSCAN16-AS1 | 0.303806034 | 3.53E-12 |
| EIF4A1 | CCDC18-AS1 | 0.5496242 | 5.76E-41 |
| CYFIP2 | CCDC18-AS1 | 0.365230259 | 2.75E-17 |
| NCBP2 | LINC02012 | 0.423976494 | 2.55E-23 |
| NCBP3 | C1RL-AS1 | 0.308812008 | 1.49E-12 |
| EIF4A1 | C1RL-AS1 | 0.505623787 | 6.16E-34 |
| NUDT16 | LINC00893 | 0.316210891 | 4.04E-13 |
| NCBP3 | LINC00893 | 0.325173866 | 7.93E-14 |
| EIF4A1 | LINC00893 | 0.559969945 | 8.95E-43 |
| CYFIP2 | ITGB2-AS1 | 0.526921684 | 3.28E-37 |
| NUDT16L1 | SRP14-AS1 | 0.330695488 | 2.83E-14 |
| NUDT7 | SRP14-AS1 | 0.306994545 | 2.04E-12 |
| CYFIP2 | SRP14-AS1 | 0.306135441 | 2.37E-12 |
| DCP2 | DBH-AS1 | 0.343675155 | 2.31E-15 |
| CYFIP2 | DBH-AS1 | 0.49251786 | 4.92E-32 |
| AGO2 | SREBF2-AS1 | 0.310175749 | 1.17E-12 |
| IFIT5 | SREBF2-AS1 | -0.30737186 | 1.91E-12 |
| NSUN2 | EXOC3-AS1 | 0.376206795 | 2.53E-18 |
| NUDT16L1 | GLIS2-AS1 | 0.458708969 | 1.73E-27 |
| NCBP1 | MIR600HG | 0.334549093 | 1.36E-14 |
| EIF4A1 | MIR600HG | 0.312754656 | 7.47E-13 |
| NUDT1 | SNHG15 | 0.396661476 | 2.30E-20 |
| SNUPN | TMEM99 | 0.335183811 | 1.21E-14 |
| TRMT112 | TMEM99 | 0.308205597 | 1.65E-12 |
| EIF4E3 | PSMB8-AS1 | 0.391177183 | 8.39E-20 |
| IFIT5 | PSMB8-AS1 | 0.435100091 | 1.33E-24 |
| CYFIP2 | PSMB8-AS1 | 0.398782831 | 1.39E-20 |
| NCBP1 | PTCSC2 | 0.40227989 | 5.98E-21 |
| DCP2 | NFYC-AS1 | 0.341764475 | 3.36E-15 |
| NCBP3 | NFYC-AS1 | 0.382402144 | 6.31E-19 |
| EIF4A1 | NFYC-AS1 | 0.469581433 | 6.77E-29 |
| CYFIP2 | NFYC-AS1 | 0.331466549 | 2.45E-14 |
| EIF4E2 | ZFAS1 | 0.310944244 | 1.03E-12 |
| EIF4G3 | ZFAS1 | -0.307696287 | 1.81E-12 |
| TRMT112 | ZFAS1 | 0.357485171 | 1.41E-16 |
| NUDT16L1 | ZNF232-AS1 | 0.360747712 | 7.11E-17 |
| NSUN2 | MIR4458HG | 0.315063902 | 4.96E-13 |
| EIF4A1 | TBILA | 0.462567605 | 5.54E-28 |
| AGO2 | TONSL-AS1 | 0.316037587 | 4.17E-13 |
| EIF4A1 | TONSL-AS1 | 0.388169728 | 1.69E-19 |
| RAMAC | CHASERR | 0.335555362 | 1.12E-14 |
| EIF4A1 | LINC-PINT | 0.474698221 | 1.42E-29 |
| RNMT | RAMP2-AS1 | 0.34826094 | 9.25E-16 |
| EIF4A1 | MIR4453HG | 0.303324572 | 3.83E-12 |
| DCP2 | STARD4-AS1 | 0.478250092 | 4.70E-30 |
| RNMT | STARD4-AS1 | 0.429149571 | 6.54E-24 |
| EIF4A1 | DICER1-AS1 | 0.315726653 | 4.41E-13 |
| CYFIP1 | OIP5-AS1 | 0.386761398 | 2.33E-19 |
| SNUPN | OIP5-AS1 | 0.438481326 | 5.29E-25 |
| NCBP2 | OR2A1-AS1 | 0.306089744 | 2.38E-12 |
| NUDT16L1 | LINC00884 | 0.354584371 | 2.56E-16 |
| NUDT3 | HCG18 | 0.441141877 | 2.55E-25 |
| NUDT16L1 | C6orf99 | 0.456819437 | 3.00E-27 |
| NUDT4 | C6orf99 | 0.349027879 | 7.93E-16 |
| EIF4A1 | SNHG12 | 0.603737956 | 3.54E-51 |
| EIF4A1 | PAN3-AS1 | 0.389476483 | 1.25E-19 |
| CYFIP2 | PAN3-AS1 | 0.30153751 | 5.18E-12 |
| RNMT | FBXL19-AS1 | 0.310829845 | 1.05E-12 |
| NUDT1 | SNHG17 | 0.307761591 | 1.79E-12 |
| EIF3D | OGFRP1 | 0.310649892 | 1.08E-12 |
| NCBP1 | DNAJC3-DT | 0.368646876 | 1.32E-17 |
| NCBP1 | FGF14-AS2 | 0.330508177 | 2.93E-14 |
| CYFIP2 | FGF14-AS2 | 0.364736027 | 3.06E-17 |
| NCBP2 | MUC20-OT1 | 0.418997245 | 9.25E-23 |
| EIF4A1 | MUC20-OT1 | 0.395839564 | 2.80E-20 |
| DCP2 | MIR155HG | 0.344139421 | 2.10E-15 |
| EIF4E3 | MIR155HG | 0.326073336 | 6.71E-14 |
| CYFIP2 | MIR155HG | 0.492907813 | 4.33E-32 |
| CYFIP2 | TRBV11-2 | 0.306014803 | 2.42E-12 |
| NUDT16L1 | RNF207-AS1 | 0.345173509 | 1.71E-15 |
| NUDT16L1 | SNHG30 | 0.327005163 | 5.65E-14 |
| RAMAC | SNHG30 | 0.309935065 | 1.22E-12 |
| NUDT16 | ZBTB11-AS1 | 0.36015893 | 8.05E-17 |
| NCBP1 | TMEM9B-AS1 | 0.330148765 | 3.14E-14 |
| NCBP2 | ACAP2-IT1 | 0.480879035 | 2.06E-30 |
| EIF4A1 | ACAP2-IT1 | 0.424454654 | 2.25E-23 |
| EIF4A1 | ADAMTSL4-AS2 | 0.417467702 | 1.37E-22 |
| CYFIP2 | FAM30A | 0.488808856 | 1.64E-31 |
| NCBP3 | LINC02604 | 0.335301753 | 1.18E-14 |
| EIF4A1 | LINC02604 | 0.487334166 | 2.64E-31 |
